# Cancer‐associated fibroblasts are associated with CD8+ T cell depletion and poor prognosis in colorectal adenocarcinoma: a multi‐omics and machine learning analysis

**DOI:** 10.1002/2056-4538.70076

**Published:** 2026-02-22

**Authors:** Myungsun Shim, One‐Zoong Kim, Byoung Kwan Son, Jung Ki Jo, Seung Wook Lee, Hong Sang Moon, Hyung Suk Kim, Mi Jung Kwon, Sung Hak Lee, Yung‐Kyun Noh, Kyueng‐Whan Min

**Affiliations:** ^1^ Department of Urology, Hanyang University Guri Hospital Hanyang University College of Medicine Guri Republic of Korea; ^2^ Department of Internal Medicine, Uijeongbu Eulji Medical Center Eulji University School of Medicine Uijeongbu Republic of Korea; ^3^ Department of Urology, College of Medicine Hanyang University Seoul Republic of Korea; ^4^ Department of Surgery, Hanyang University Guri Hospital Hanyang University College of Medicine Guri Republic of Korea; ^5^ Department of Pathology, Hallym University Sacred Heart Hospital Hallym University College of Medicine Anyang Republic of Korea; ^6^ Department of Hospital Pathology, Seoul St. Mary's Hospital, College of Medicine The Catholic University of Korea Seoul Republic of Korea; ^7^ Department of Computer Science Hanyang University Seoul Republic of Korea; ^8^ School of Computational Sciences Korea Institute for Advanced Study Seoul Republic of Korea; ^9^ Department of Pathology, Uijeongbu Eulji Medical Center Eulji University School of Medicine Uijeongbu Republic of Korea

**Keywords:** fibroblast, carcinoma, colon, prognosis, lymphocytes, machine learning

## Abstract

Fibroblastic proliferation in various tumor microenvironments influences cancer survival through complex interactions with diverse immune responses. This study investigated the impact of histologically unique activated cancer‐associated fibroblasts (aCAFs) on survival outcomes and immune responses and examined their association with various pathophysiological mechanisms. We analyzed a total of 1,024 colorectal adenocarcinoma patients from two cohorts. aCAFs were evaluated based on hematoxylin and eosin‐stained whole‐slide images, and their associations with clinicopathological features, immune cell infiltration, and survival were assessed. We developed a machine learning‐based survival prediction model incorporating aCAFs and clinicopathologic parameters. Additionally, we performed differential gene expression analysis, functional enrichment analyses, and *in vitro* drug screening of aCAF‐related genes. aCAFs were associated with advanced T stage, lymphovascular invasion, perineural invasion, and decreased CD8+ and CD4+ T cell infiltration. aCAFs were also associated with worse overall and disease‐free survival in both univariate and multivariate analyses. Functional enrichment analysis revealed that aCAF‐related genes were implicated in immunosuppressive signaling, oxidative stress regulation, and tumor progression pathways. Survival prediction models based on machine learning and incorporating aCAFs demonstrated superior prognostic accuracy for overall survival and disease‐free survival compared to models excluding aCAFs. Our analysis of aCAFs' association with immune responses through bioinformatics‐based genomic analysis and machine learning provides a foundation for future research in CRC patients.

## Introduction

Colorectal adenocarcinoma (CRC) represents a significant global health burden, with rising incidence and mortality rates influenced by genetic predisposition, environmental factors, and lifestyle choices [1]. Despite significant advancements in surgical techniques, chemotherapy, and immunotherapy, patient outcomes remain suboptimal, particularly in advanced‐stage disease. A key determinant of CRC progression is the tumor microenvironment (TME), which plays a crucial role in immune evasion and therapeutic resistance [2]. Among the immune components, CD8+ T cells are central to antitumor immunity, as they mediate cytotoxic responses against malignant cells. The immunosuppressive nature of the TME in CRC often results in impaired CD8+ T cell infiltration and function, thereby facilitating disease progression and resistance to therapy [3].

Cancer‐associated fibroblasts (CAFs) represent a critical stromal component within the CRC microenvironment, playing a pivotal role in tumor progression and immune modulation [4]. These cells have been shown to promote tumor proliferation, invasion, and metastasis through extracellular matrix remodeling and the secretion of immunosuppressive cytokines. In the context of cancer progression, CAFs have been observed to impede immune cell function through the action of transforming growth factor‐beta (TGF‐β) and other immunosuppressive mediators [5], while concomitantly inducing LRG1 expression in tumor cells *via* the STAT3 pathway, thus promoting processes such as epithelial–mesenchymal transition, migration, and invasion [6].

Recent advancements in single‐cell RNA sequencing have enabled the extensive classification of CAF phenotypes, including myoblast‐like CAFs (myCAFs), inflammatory CAFs (iCAFs), and antigen‐presenting CAFs (apCAFs). These classifications are based on marker genes, biological functions, spatial distribution within the TME, and cellular interactions [7]. Recent research has identified additional subpopulations, including senescent CAFs. These cells are distinguished by their senescence‐associated secretory phenotype (SASP), which is characterized by the expression of specific SASP‐related genes, such as interleukins (IL‐6, IL‐8), chemokines (CCL2, CXCL1), and matrix metalloproteinases (MMPs) [8, 9]. However, these classifications are limited by their molecular phenotyping approach and lack intuitive histopathological features for identification.

The present study employs classical approaches to identify histologically distinctive activated cancer‐associated fibroblasts (aCAFs) through direct examination of hematoxylin and eosin (H&E)‐stained whole slide images. This approach aims to investigate their clinical significance and diverse biological implications beyond the molecularly classified CAF subtypes in CRC. In this study, an integrated analysis was employed to evaluate the potential impact of aCAFs on disease progression and immune response. This analysis combined histopathological examination, large‐scale genomic data, bioinformatics, and machine learning approaches (supplementary material, Figure S1).

## Materials and methods

### Patient selection

This study included 404 patients with surgically resected CRC (305 colon, 99 rectum) treated between 2000 and 2010 at hospitals affiliated with Eulji Medical Center. Clinicopathological parameters collected included patient age, sex, tumor location, histological grade, pathological stage according to the 8th edition of the American Joint Committee on Cancer (AJCC) staging system, lymphovascular invasion (LVI), perineural invasion (PNI), and tumor necrosis. The study protocol received approval from the Institutional Review Board of Uijeongbu Eulji University Hospital (IRB number: 2023‐12‐005‐006), which covers hospitals affiliated with Eulji Medical Center, and adhered to the ethical standards outlined in the Declaration of Helsinki, revised in 2008. Due to the retrospective nature of the study and the anonymized data used, informed consent was not obtained from individual participants. The IRB granted permission to waive the requirement for documented informed consent.

For validation, we obtained data from The Cancer Genome Atlas (TCGA) dataset (https://gdc.cancer.gov/about-data/publications/pancanatlas) (https://portal.gdc.cancer.gov/) comprising 620 CRC cases (449 colon, 171 rectum) with available clinicopathological information [10, 11, 12].

### Evaluation of cancer associated fibroblasts and immunohistochemistry

We evaluated aCAFs within stromal components using H&E‐stained whole‐slide images from both our cohort and the TCGA dataset.

**Figure 1:**
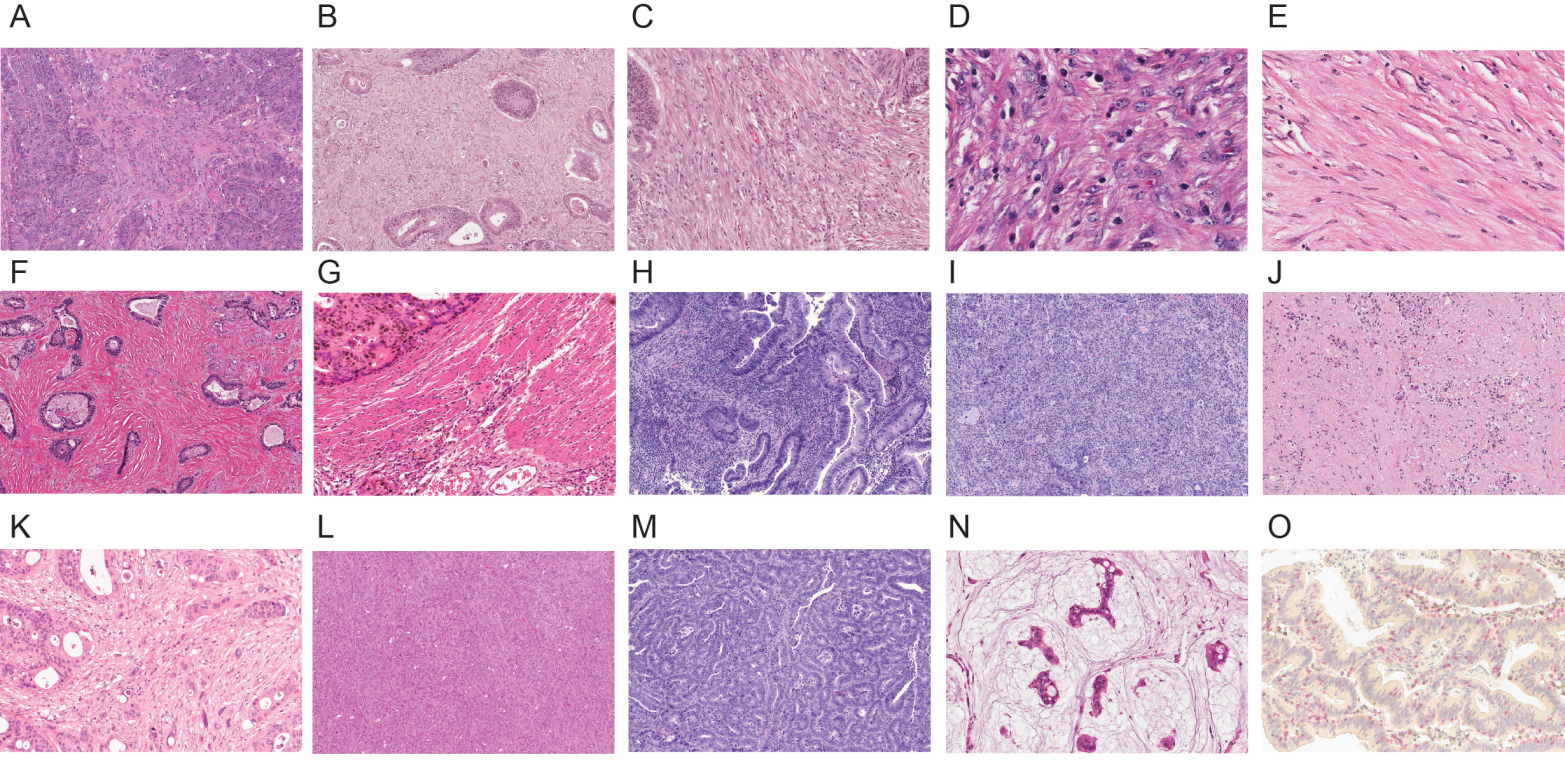
Histopathological characterization of the colorectal adenocarcinoma microenvironment representative microscopic images demonstrating: (A, B) Activated cancer‐associated fibroblasts (aCAFs) infiltrating between tumor cells (×200). (C) Higher magnification view of aCAFs traversing tumor cell clusters (×400). (D) High‐power view of aCAFs demonstrating large, oval, euchromatic nuclei with prominent nucleoli (×1,000). (E) Mature cancer‐associated fibroblasts exhibiting flattened morphology with small, hyperchromatic nuclei (×1,000). (F) Collagen fiber bundle deposition within the tumor stroma (×200). (G) Adjacent proper muscle tissue (×200). (H) Tumor‐infiltrating lymphocytes (TILs) (×200). (I) Neutrophilic abscess formation (×200). (J) Area of tumor necrosis (×200). (K) Spindle‐shaped tumor cell nuclei mimicking aCAF morphology (×400). (L) Large tumor cell aggregates lacking stromal components (×200). (M) Representative area of stroma‐poor tumor (×200). (N) Tumor cell clusters suspended within mucin pools (×400). (O) Immunohistochemical dual staining demonstrating CD8+ T cells (red) and CD4+ T cells (brown) (×400). All magnifications indicated represent total magnification (objective × eyepiece).

aCAFs were identified based on characteristic morphological features including spindle‐shaped cell morphology with large, plump nuclei exhibiting irregular contours, prominent nucleoli, coarse and heterogeneous chromatin distribution, and elevated nuclear‐to‐cytoplasmic ratios (Figure 1). Representative high‐power images demonstrating these cytologic features of activated CAFs are shown in Figure 1C, D. In contrast, Figure 1E presents a high‐power image of inactivated CAFs, illustrating the distinct cytologic differences between activated and mature stromal fibroblasts. The remaining images demonstrate the architectural distribution of CAFs within the tumor stroma at lower magnification (Figure 1A, B, F–N). Inactivated CAFs displayed a monomorphic spindle morphology characterized by diminutive, thin, wavy, and slender nuclei with pale chromatin, indistinct nucleoli, and reduced nuclear‐to‐cytoplasmic ratios.

To ensure accurate identification, the following structures were excluded from aCAF assessment: (1) dense collagen fiber bundles, (2) mature fibroblasts with typical quiescent morphology, (3) thick collagen bundles, (4) smooth muscle tissue, (5) clusters of inflammatory cells including tumor‐infiltrating lymphocytes (TILs) and neutrophils, and (6) mucin pools.

Three pathologists (K‐WM, MJK, YCW), blinded to clinical and outcome data, independently evaluated each case by systematically assessing 10 representative high‐density cancer fields at ×100 magnification. These fields encompassed both the tumor mass center and the invasive front, including intratumoral and peritumoral stromal regions. A tumor was classified as aCAF‐positive when >10% of stromal cells across the evaluated fields demonstrated the aforementioned morphological features of fibroblasts. This semiquantitative threshold was established based on CAF assessment methodologies validated in other solid organ malignancies and was refined through consensus review among the three pathologists to ensure reproducibility despite inherent variability in stromal composition and tumor sampling areas [13, 14, 15].

A quantitative analysis of TILs was conducted at the leading edge of tumor invasion and within the intratumoral region, employing a high‐power field (original magnification ×400). Immunostaining for anti‐CD8 (clone 4B11, Leica Biosystems, Newcastle, UK) and anti‐CD4 (clone 4B12, Leica Biosystems, Newcastle, UK) was performed using the Bond Polymer Refine Detection System (Leica Biosystems, Newcastle Ltd., Newcastle, UK) according to the manufacturer's instructions (Figure 1O).

A comparative analysis of marker gene expression was conducted using RNA sequencing (RNA‐seq) data across three well‐characterized molecular subtypes of CAFs: myCAFs characterized by *ACTA2*, *TAGLN*, *MMP11*, *HOPX*, *POSTN*, *TPM1*, and *TPM2* expression; iCAFs defined by *IL6*, *IL8*, *PDGFRA*, *CXCL1*, *CXCL2*, *CXCL12*, *CCL2*, *CFD*, *DPT*, *LMNA*, *AGTR1*, and *HAS1* markers; and apCAFs distinguished by *CD74*, *SLPI*, *SAA3P*, *HLA‐DRA*, *HLA‐DRB1*, and *CIITA* expression [16, 17]. Based on this established molecular framework, we conducted a comprehensive evaluation of the molecular characteristics and gene expression patterns of morphologically identified aCAFs.

The TCGA cases were further stratified into two groups: senescent cancer‐associated fibroblasts (sCAFs; *n* = 22) and non‐senescent cancer‐associated fibroblasts (nsCAFs) (*n* = 120). This stratification was based on the expression of SASP‐related factors. The identified factors encompassed inflammatory cytokines (IL‐6, IL‐8) [18, 19] growth factors (VEGFA) [20, 21] chemokines (CCL2) [22] and proteases (MMP‐1, TIMP‐1) [20, 21]. Cases were classified as SASP‐positive when they showed high expression (above the mean values) in four or more of these six factors.

### Functional enrichment analysis, *in silico* cytometry, and immune dysfunction

For biological interpretation, we investigated the Gene Ontology (GO) and the associations between various diseases and aCAF‐linked gene clusters by analyzing functional similarities across 620 cases of CRC from the TCGA dataset containing 20,514 mRNA sequences. We analyzed differentially expressed genes (DEGs) between patients with and without aCAFs using the EnhancedVolcano tool (https://github.com/kevinblighe/EnhancedVolcano). DEGs were identified using threshold criteria of *p* value <0.05 and false discovery rate <0.05. For semantic similarity analysis, we employed DOSE, an R/Bioconductor package that enables comprehensive exploration of molecular functions and their associated genes [23]. We conducted pathway analysis using three curated and peer‐reviewed databases: KEGG pathways (https://www.genome.jp/kegg), WikiPathways (data.wikipathways.org), and CellMarker (http://bio-bigdata.hrbmu.edu.cn/CellMarker) [24], allowing for thorough examination of molecular pathways [25].

In the TCGA dataset, we employed *in silico* cytometry using CIBERSORT for the analysis of leukocyte subsets [11]. Furthermore, we utilized the Tumor Immune Dysfunction and Exclusion (TIDE) tool to evaluate immune cell dysfunction [26].

### Single‐cell RNA‐seq data processing and analysis

We obtained the processed single‐cell RNA‐seq dataset (GSE200997) from the Gene Expression Omnibus (GEO), which includes colorectal cancer tissues (*n* = 16) and paired normal samples (*n* = 7) [27]. The annotation file containing cell barcodes and metadata was loaded, and the raw Unique Molecular Identifier (UMI) count matrix was imported. Data preprocessing and downstream analyses were performed using the Seurat package (v4.0). Raw UMI counts were converted into a Seurat object with the following parameters: min.cells = 3 and min.features = 200. Normalization, variable feature selection (2,000 features), scaling, principal component analysis (PCA), and UMAP dimensionality reduction were subsequently applied. Cell clusters were annotated based on canonical marker expression: epithelial/tumor cells, stromal cells, and immune cells. For visualization, we generated UMAP plots to display cell‐type distributions in normal and tumor tissues. *ACTA2* (Actin Alpha 2, α‐SMA) expression was further evaluated, and relative expression across major cell types was quantified.

### Machine learning algorithms

We constructed predictive survival models using two machine learning (ML) algorithms: adaptive elastic net (AEN) based on linear regression [incorporating both lasso (L1) and ridge (L2) penalties] and gradient boosting machine (GBM) based on decision trees [28]. A survival prediction model was developed by integrating aCAFs with clinicopathological parameters, including T stage, age, sex, histological grade, lymphovascular invasion, and perineural invasion. The ML algorithms were applied to 404 cases of CRC from our cohort, which was stratified into training (70%) and validation (30%) sets [29].

In the case of the AEN, regularization was balanced using an alpha value of 0.5. The regularization strength was optimized through 10‐fold cross‐validation. Survival models were constructed by incorporating candidate genes to predict status (death or recurrence) and survival period. Model parsimony was achieved through variable selection, a process that involved the elimination of parameters with negligible weights. The performance of the model was evaluated using both nomograms and time‐dependent receiver operating characteristic (ROC) curves.

For the AEN, performance optimization was achieved by balancing lasso and ridge regularization with an alpha value of 0.5. The regularization strength was determined through 10‐fold cross‐validation, ensuring a balanced representation of aCAFs in each fold. The GBM implementation utilized a learning algorithm that autonomously selected and combined multiple covariates based on multivariate Bernoulli models. Hyperparameter optimization, including learning rate adjustment, was performed for each combination of selected covariates and learning algorithm using grid‐search cross‐validation across predefined ranges. The final optimal models were subsequently trained using the selected covariates and optimized hyperparameters. Model performance for the GBM algorithm was evaluated using ROC curves. To assess the contribution of each candidate gene to the survival prediction models (affecting survival probability and Cox risk score values), we utilized SHapley Additive exPlanation (SHAP) [30].

### Statistical analysis

Statistical analyses were performed as follows: correlations between clinicopathological parameters and aCAF presence were evaluated using the *χ* ^2^ test, while continuous variables were compared using Student's *t*‐test. Survival analyses were conducted using the Kaplan–Meier method with log‐rank test comparisons. Independent prognostic markers for survival were identified through multivariate Cox regression analyses. Inter‐observer reproducibility for aCAF assessment was evaluated using Fleiss’ κ statistics, which quantifies the degree of agreement among multiple raters beyond chance alone [31]. Statistical significance was defined as a two‐sided *p* value <0.05. All analyses were performed using R and SPSS Statistics software (version 25, IBM Corporation, Armonk, NY, USA).

## Results

### Inter‐observer reproducibility of aCAF assessment

To validate the robustness of morphological criteria for aCAF identification, inter‐observer agreement among three independent pathologists was quantified using Fleiss' *κ* statistics. In the institutional cohort (*n* = 404), substantial agreement was achieved with *κ* = 0.775 (*p* < 0.001) (supplementary material, Table S1). External validation in the TCGA cohort (*n* = 620) demonstrated almost perfect agreement with *κ* = 0.82 (*p* < 0.001) (supplementary material, Table S2). These findings confirm that semiquantitative identification of aCAFs based on defined morphological criteria is highly reproducible across independent observers and geographically distinct patient populations.

### Clinicopathological characteristics and survival outcomes associated with aCAFs and sCAFs

**Figure 2:**
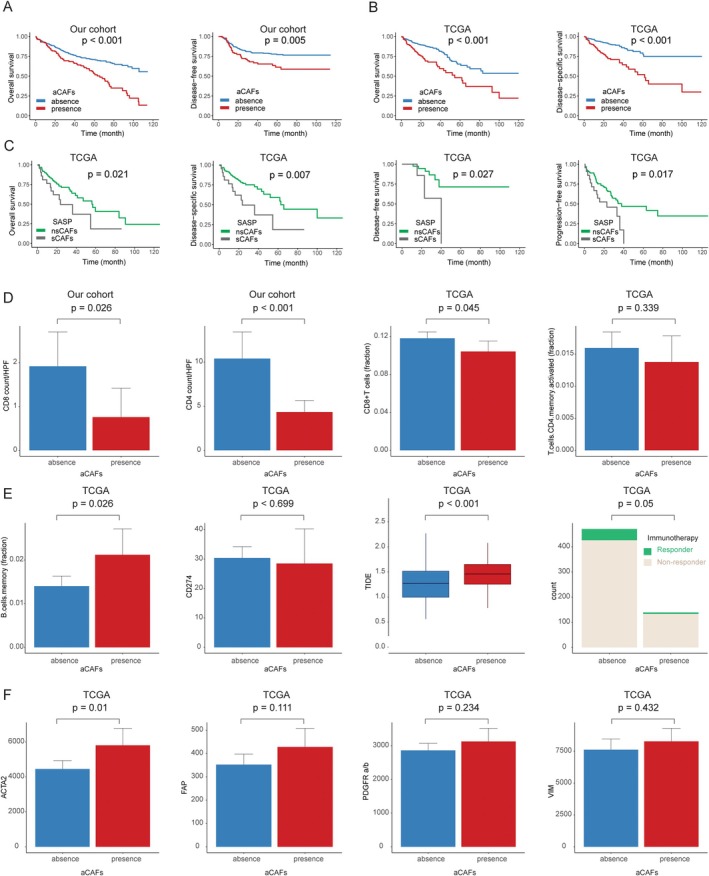
Survival outcomes and immune profile analysis. (A) Kaplan–Meier analysis of overall survival and disease‐free survival stratified by activated cancer‐associated fibroblast (aCAF) status in the study cohort. (B) Overall survival and disease‐specific survival curves from The Cancer Genome Atlas (TCGA) cohort stratified by aCAF presence. (C) Survival analyses (overall, disease‐specific, disease‐free, and progression‐free survival) in the TCGA cohort comparing non‐senescent CAFs (nsCAFs) versus senescent CAFs (sCAFs). (D) Comparative analyses of CD4+ and CD8+ T cell densities per high‐power field (HPF) between aCAF‐positive and aCAF‐negative groups in our cohort, with corresponding CD8+ T cell and memory CD4+ T cell fractions in the TCGA dataset. (E) Distribution of memory B cell fractions, CD274 (PD‐L1 coding gene) expression levels, tumor immune dysfunction and exclusion (TIDE) scores, and immunotherapy response patterns in the TCGA dataset. (F) Expression profiles of aCAF‐associated biomarkers, including *ACTA2* (α‐smooth muscle actin‐encoding gene), *FAP* (fibroblast activation protein‐α), *PDGFRA/PDGFRB* (platelet‐derived growth factor receptor α/β), and *VIM* (vimentin‐encoding gene) in the TCGA.

Among the 404 patients analyzed in our cohort, aCAFs were identified in 99 cases (24.5%), while 305 cases (75.5%) were aCAF‐negative. The presence of aCAFs was significantly associated with adverse clinicopathological features, including advanced age, higher T and N stages, high histological grade, LVI, PNI, and tumor necrosis (all *p* < 0.05) (Table 1). Kaplan–Meier survival analyses revealed that patients with aCAF‐positive tumors demonstrated significantly inferior disease‐free survival (DFS) (*p* = 0.005) and overall survival (OS) (*p* < 0.001) compared to aCAF‐negative cases (Figure 2A). These associations persisted in multivariate Cox regression analysis after adjusting for age, sex, T stage, histological grade, LVI, PNI, and tumor necrosis (DFS: *p* = 0.042; OS: *p* = 0.001) (Table 2).

**Table 1:** Correlation between clinicopathological parameters and activated cancer‐associated fibroblasts in our cohort (404 cases)

**Parameter**	Activated cancer‐associated fibroblasts		*p* value *χ* ^2^
	Absence (*n* = 305), *n* (%)	Presence (*n* = 99), *n* (%)	
Age (years)	62.4 ± 11.3	66.6 ± 11.2	0.001*
Tumor size (cm)	5.0 ± 2.2	5.0 ± 2.0	0.959*
Sex			
Female	118 (38.7)	40 (40.4)	0.853
Male	187 (61.3)	59 (59.6)	
T stage			
1	31 (10.2)	4 (4.0)	0.041^†^
2	35 (11.5)	5 (5.1)	
3	186 (61.0)	67 (67.7)	
4	53 (17.4)	23 (23.2)	
N stage			
0	148 (48.5)	36 (36.4)	0.026^†^
1	76 (24.9)	23 (23.2)	
2	81 (26.6)	40 (40.4)	
Location			
Cecum to sigmoid	196 (64.3)	59 (59.6)	0.403
Rectosigmoid to rectum	109 (35.7)	40 (40.4)	
Histological grade			
Well differentiated	29 (9.5)	2 (2.0)	0.015^‡^
Moderately differentiated	136 (44.6)	52 (52.5)	
Poorly differentiated	140 (45.9)	45 (45.5)	
Lymphovascular invasion			
Negative	141 (46.2)	31 (31.3)	0.013
Positive	164 (53.8)	68 (68.7)	
Perineural invasion			
Negative	166 (54.4)	41 (41.4)	0.033
Positive	139 (45.6)	58 (58.6)	
Tumor necrosis			
Negative	137 (45.5)	20 (20.2)	<0.001
Positive	164 (54.5)	79 (79.8)	

T or N stage by the 8th edition of the American Joint Committee on Cancer.

* Student's *t*‐test.

^†^ Linear‐by‐linear association.

^‡^ Well differentiated versus moderately differentiated and poorly differentiated.

**Table 2:** Disease‐free survival and overall survival analyses according to activated cancer‐associated fibroblasts in our cohort (404 cases)

	Univariate*	Multivariate^†^	HR	95% CI	
Disease‐free survival					
aCAFs (absence versus presence)	0.005	0.042	1.600	1.016	2.518
Age (≤55 versus >55)	0.653	0.570	0.866	0.526	1.424
Sex (women versus men)	0.016	0.008	1.876	1.175	2.993
T stage (1, 2 versus 3, 4)	<0.001	0.025	2.913	1.142	7.430
Histological grade (1, 2 versus 3)	<0.001	0.007	1.832	1.182	2.842
LVI (absence versus presence)	<0.001	0.008	2.145	1.221	3.770
PNI (absence versus presence)	<0.001	0.353	1.262	0.772	2.062
Necrosis (absence versus presence)	0.134	0.923	1.023	0.644	1.626
Overall survival					
aCAFs (absence versus presence)	<0.001	0.001	1.738	1.237	2.443
Age (≤55 versus >55)	0.002	0.011	1.847	1.149	2.971
Sex (women men)	0.019	0.025	1.489	1.052	2.108
T stage (1, 2 versus 3, 4)	<0.001	0.019	2.068	1.125	3.800
Histological grade (1, 2 versus 3)	0.001	0.018	1.486	1.071	2.063
LVI (absence versus presence)	<0.001	0.256	1.258	0.847	1.869
PNI (absence versus presence)	<0.001	0.098	1.379	0.943	2.018
Necrosis (absence versus presence)	0.120	0.923	1.018	0.714	1.449

T stage by the 8th edition of the American Joint Committee on Cancer. Histological grade: 1 (well‐differentiated), 2 (moderately differentiated), 3 (poorly differentiated).

aCAFs, activated cancer‐associated fibroblasts; LVI, lymphovascular invasion; PNI, perineural invasion.

* Log rank test.

^†^ Cox proportional hazard model.

To validate the clinicopathological significance of aCAFs in an independent cohort, we analyzed data from TCGA CRC dataset (*n* = 617). Consistent with our institutional cohort, aCAF‐positive tumors in the TCGA cohort were significantly associated with advanced T stage, higher N stage, LVI, PNI, and prominent tumor necrosis (all *p* < 0.05), though no significant differences were observed in patient age or tumor size between groups (supplementary material, Table S3). Survival analysis confirmed that aCAF positivity was significantly associated with reduced disease‐specific survival (DSS) and OS (both *p* < 0.001) (Figure 2B). In multivariate Cox regression analysis, aCAFs remained an independent adverse prognostic factor for both DSS and OS (both *p* < 0.001) (supplementary material, Table S4).

Among the 142 TCGA cases with aCAF‐positive tumors, the subset demonstrating sCAFs exhibited significantly worse clinical outcomes compared to those with nsCAFs, including decreased OS (*p* = 0.021), DSS (*p* = 0.007), DFS (*p* = 0.027), and progression‐free survival (PFS) (*p* = 0.017) (Figure 2C).

### Characteristics of aCAFs and associated immune response

Analysis of immune parameters revealed significant correlations between the presence of aCAFs and reduced infiltration of both CD8+ T cells and CD4+ T cells (*p* = 0.026 and <0.001, respectively) in our cohort. These findings were corroborated in the TCGA dataset, where the presence of aCAFs significantly correlated with decreased CD8+ T cell fraction, reduced memory B cell fraction, and elevated TIDE scores (all *p* < 0.05). Furthermore, patients with aCAFs demonstrated a higher frequency of non‐response to immunotherapy compared to those without aCAFs (p = 0.05). Among established aCAF‐associated biomarkers (*ACTA2*, *FAP*, *PDGFR‐α/β*, and *VIM*), *ACTA2*, which encodes α‐smooth muscle actin, showed significantly elevated expression in aCAF‐positive patients (*p* = 0.01) (Figure 2D–F).

**Figure 3:**
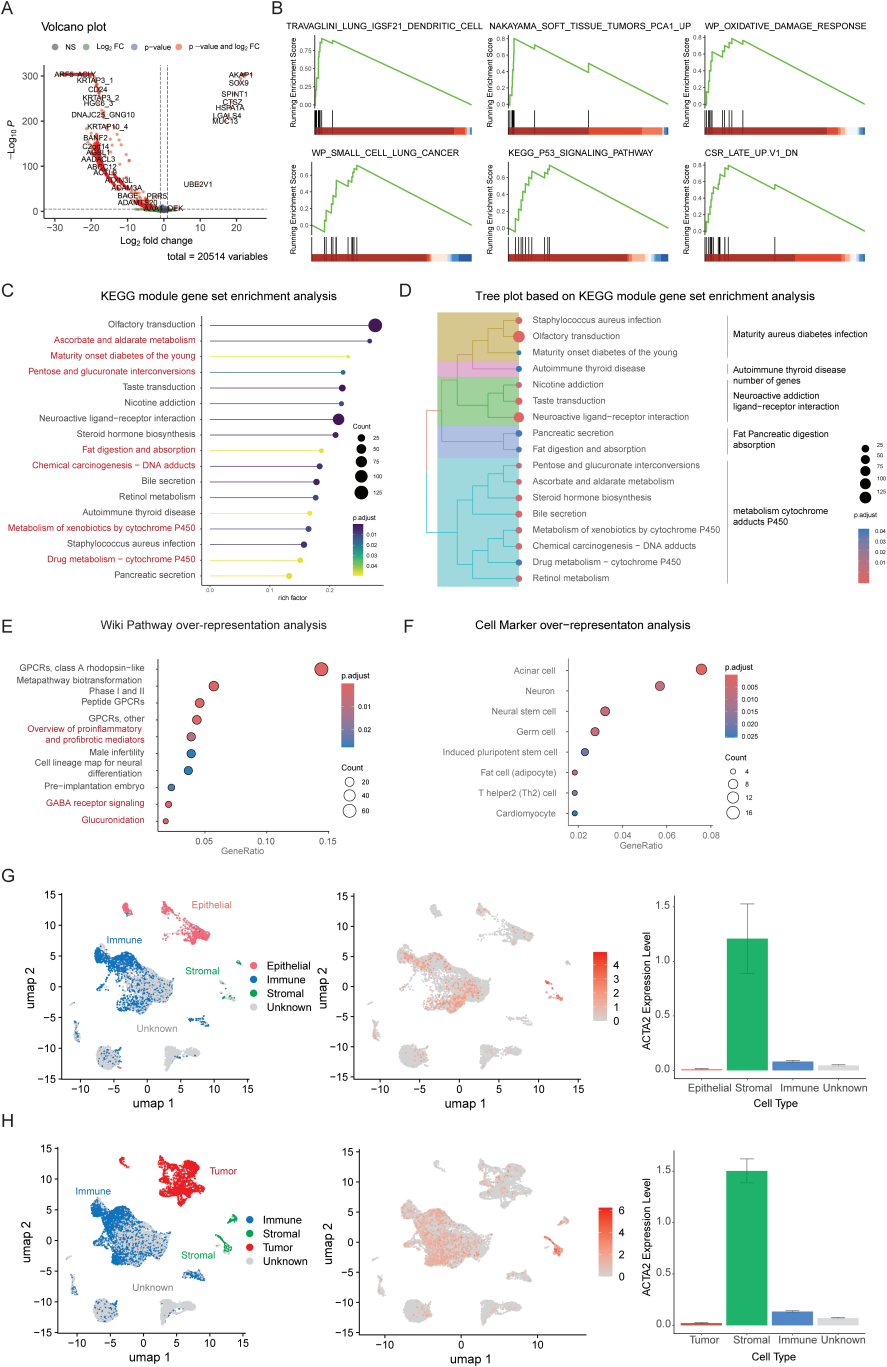
Bioinformatic analysis of activated cancer‐associated fibroblasts (aCAFs)‐associated molecular signatures. (A) Differential gene expression analysis revealing aCAFs‐specific transcriptional signatures. (B) Gene set enrichment analysis (GSEA) using MSigDB, highlighting significant pathways. (C, D) Functional enrichment analysis and hierarchical tree plot based on the Kyoto Encyclopedia of Genes and Genomes (KEGG) pathways. (E) Over‐representation analysis utilizing Wiki Pathways database. (F) Cell type–specific over‐representation analysis based on CellMarker database. (G) Distribution and expression of *ACTA2* in normal and tumor colorectal tissues. UMAP visualization of single‐cell populations from normal colorectal tissue, classified into epithelial, immune, stromal, and unknown groups (left). *ACTA2* expression is shown in red, primarily localized to stromal cells (middle). Bar plot indicates significantly higher expression of *ACTA2* in stromal cells compared to other cell types (right). (H) UMAP plots of colorectal cancer tissue showing tumor, immune, stromal, and unknown cell populations (left). *ACTA2* expression is enriched in stromal cells with minor expression in tumor and immune compartments (middle). Bar plot demonstrates an overall increase in *ACTA2* expression in tumor‐associated stromal cells compared to stromal cells in normal tissue (right).

Transcriptomic analysis of the TCGA dataset, encompassing RNA sequencing data from 620 patients with 20,514 genes, was performed to identify DEGs between patients stratified by aCAFs status. Among the total gene set, 4,859 genes demonstrated significant differential expression, with 1,210 genes showing upregulation and 3,649 showing downregulation (Figure 3A). Gene Set Enrichment Analysis (GSEA) identified several significantly enriched biological pathways. The analysis showed enrichment of gene signatures associated with dendritic cell markers (TRAVAGLINI LUNG IGSF21 DENDRITIC CELL), soft tissue tumor progression (NAKAYAMA SOFT TISSUE TUMORS PCA1 UP), and cellular responses to oxidative stress (WP OXIDATIVE DAMAGE RESPONSE). Additionally, there was significant enrichment of cancer‐related pathways, including small cell lung cancer signatures (WP SMALL CELL LUNG CANCER) and p53 signaling pathway components (KEGG P53 SIGNALING PATHWAY). The analysis also revealed enrichment of genes downregulated in the late cellular stress response (CSR LATE UP.V1 DN). These findings suggest potential mechanistic links between activated cancer‐associated fibroblasts and multiple cancer‐promoting and stress‐response pathways (Figure 3B).

A Kyoto Encyclopedia of Genes and Genomes (KEGG) module analysis revealed an overrepresentation of aCAFs‐related genes in ascorbate and aldarate metabolism, chemical carcinogenesis via DNA adducts, and drug metabolism (Figure 3C, D). Wiki Pathway over‐representation analysis demonstrated significant enrichment of aCAF‐related genes in multiple signaling cascades, including proinflammatory and profibrotic mediator pathways, GABA receptor signaling, and glucuronidation pathways (Figure 3E). CellMarker over‐representation analysis identified significant enrichment of aCAFs‐related genes in markers characteristic of diverse cell populations, notably adipocytes, neurons, and Th2 cells (Figure 3F).

Gene expression analysis of aCAFs revealed significantly higher levels of *ACTA2*, *TAGLN*, *MMP11*, *HOPX*, *POSTN*, *TPM1*, and *TPM2* (all *p* < 0.05), which is consistent with the molecular signatures of myCAFs rather than iCAFs or apCAFs. However, no significant differences were observed for iCAFs or apCAF markers (data not shown).

To investigate the cellular distribution of *ACTA2* expression, we analyzed scRNA‐seq data from the GSE200997 dataset. In normal colorectal tissue, cell clusters were classified into epithelial, immune, and stromal groups. *ACTA2* expression was predominantly observed in stromal cells, with minimal expression in epithelial and immune populations (Figure 3G). In colorectal cancer tissue, cell clusters were categorized into tumor, immune, and stromal compartments. Consistent with normal tissue, *ACTA2* was mainly expressed in stromal cells, with low‐level expression detected in tumor and immune subsets (Figure 3H). Notably, the overall expression level of *ACTA2* was slightly elevated in stromal cells from tumor tissue compared to normal stromal cells.

### Survival prediction of aCAFs using machine learning

**Figure 4:**
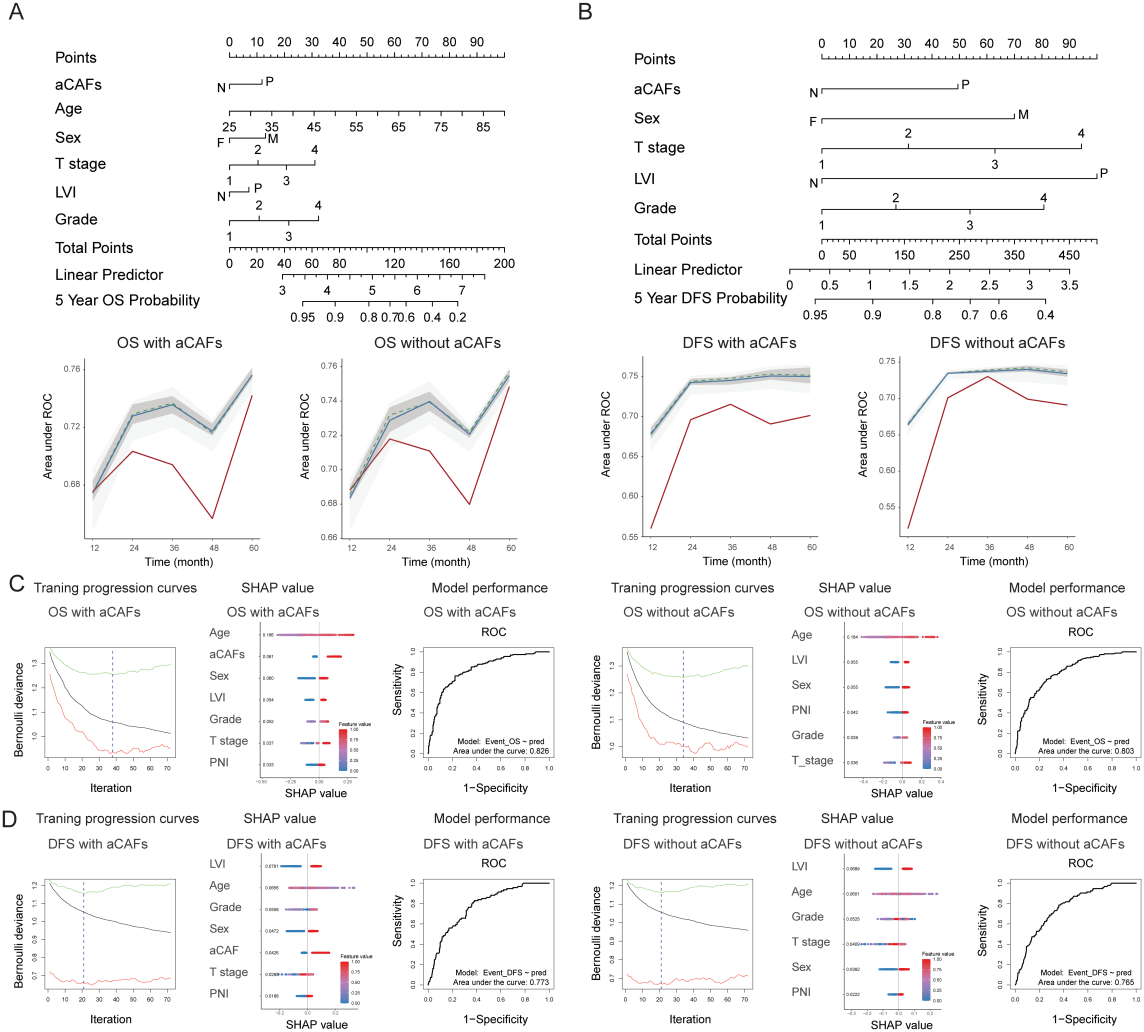
Predictive Modeling and Drug Response Analysis. (A) Nomogram for overall survival prediction using adaptive elastic net regression (upper panel) and time‐dependent ROC curve comparison of models with and without activated cancer‐associated fibroblasts (aCAFs) parameters (lower panels). (B) Nomogram for disease‐free survival prediction using adaptive elastic net regression (upper panel) and time‐dependent ROC curve comparison of models with and without aCAFs parameters (lower panels). (C, D) Development and validation of gradient boosting machine learning models with and without aCAF parameters. Training progression curves comparing aCAF‐inclusive and aCAF‐exclusive models. SHAP (SHapley Additive exPlanations) value analysis demonstrating the relative contribution of individual parameters to overall survival (OS) and disease‐free survival (DFS) prediction. Model performance assessment using receiver operating characteristic (ROC) curves based on multivariate Bernoulli distribution.

A survival prediction model was developed using the AEN, incorporating aCAFs status, age, sex, T stage, lymphovascular invasion, and histological grade. During the machine learning process, while the ‘age’ factor was excluded from the DFS nomogram, it remained a significant component in the OS nomogram. For the OS prediction (5‐year survival), the model incorporating aCAFs demonstrated superior performance with a mean area under the curve (AUC) of 0.756 (range: 0.674–0.756) in the training set and 0.742 (range: 0.675–0.742) in the validation set, compared to the model without aCAFs, which achieved AUCs of 0.755 (range: 0.683–0.755) and 0.748 (range: 0.688–0.748) in the training and validation sets, respectively (Figure 4A). Similarly, for DFS prediction, the aCAFs‐inclusive model showed enhanced performance with mean AUCs of 0.750 (range: 0.678–0.751) in the training set and 0.701 (range: 0.560–0.715) in the validation set, surpassing the model without aCAFs, which achieved AUCs of 0.734 (range: 0.663–0.739) and 0.690 (range: 0.521–0.730) in the training and validation sets, respectively (Figure 4B).

The survival prediction models using the GBM further confirmed the superior predictive capability of models that incorporate aCAFs. The OS model incorporating aCAFs achieved a mean AUC of 0.826, compared to 0.803 for the model without aCAFs (Figure 4C). This pattern was consistently observed in the DFS model, where the aCAFs‐inclusive model achieved a mean AUC of 0.773 compared to 0.765 for the model without aCAFs. Among all clinicopathological variables analyzed, age emerged as the strongest predictor for OS, while lymphovascular invasion demonstrated the highest predictive value for DFS (Figure 4D).

## Discussion

This study diverges from previous research in that it focuses on aCAFs, which exhibit distinct histopathological findings among intra‐ and peritumoral stromal components. A critical consideration in establishing such morphology‐based CAF classification is the reproducibility of histological assessment. In this study, aCAF identification was performed independently by three board‐certified pathologists blinded to clinical outcomes, and Fleiss' *κ* analysis demonstrated substantial inter‐observer agreement in both cohorts. The 10% threshold for aCAF positivity, derived from established semiquantitative CAF assessment methods, showed high reproducibility and consistent associations with adverse clinical outcomes, validating its practical utility despite the inherent semiquantitative nature of stromal evaluation.

Based on this reproducible histopathological classification, we investigated the associations between aCAF status and clinical outcomes as well as immune responses in CRC. aCAFs were associated with unfavorable outcomes, including reduced CD8+ T‐cell infiltration, elevated TIDE scores, and an impaired response to immunotherapy, findings that were validated in two independent cohorts. Furthermore, SASP‐based stratification revealed that sCAFs were linked to worse survival compared to nsCAFs.

CAFs exert immunosuppressive effects through multiple mechanisms. Previous studies have demonstrated that CAFs secrete cytokines such as TGF‐β and IL‐6, which inhibit CD8+ T‐cell recruitment and activation while fostering an immunosuppressive microenvironment [32]. Additionally, CAFs contribute to extracellular matrix remodeling, creating physical barriers that impede immune cell infiltration [33]. Recent studies in breast cancer models have demonstrated that CAF‐rich tumors generate an immunologically cold microenvironment by excluding CD8+ T‐cells, mediated in part by the CAF receptor Endo180 (MRC2), which is predominantly expressed on myCAFs [34]. Deletion of MRC2 reduces αSMA‐expressing CAFs, promotes CD8+ T‐cell infiltration, and enhances responses to immune checkpoint blockade. Consistent with these findings, high MRC2 expression in human tumors correlates with poor response to anti‐PD‐1 therapy. Additional mechanisms include FAP+ CAF‐derived CXCL12 and stromal TGF‐β signaling, both of which restrict CD8+ T‐cell infiltration and attenuate responses to checkpoint blockade [35, 36]. In our study, aCAF presence was associated with reduced CD8+ T‐cell infiltration and elevated TIDE scores, consistent with these reported immunosuppressive mechanisms and supporting their role in resistance to immunotherapy.

The molecular characterization of morphologically identified aCAFs in our study revealed signatures closely resembling myCAFs [37]. These cells, characterized by high nuclear‐to‐cytoplasmic ratios and prominent nucleoli resembling primitive immature fibroblasts, exhibited substantial upregulation of ACTA2 (α‐SMA), a pivotal regulator of immunosuppression and tumor progression [38]. Although ACTA2 is traditionally regarded as a canonical marker of myofibroblasts, emerging evidence indicates that carcinoma cells undergoing TGF‐β‐driven epithelial‐mesenchymal transition can also upregulate ACTA2 [39]. This upregulation enhances contractile activity [40], facilitating mechanical activation of latent TGF‐β1 through integrin‐mediated pathways [41]. The contractile force generated by α‐SMA‐positive cells thus plays a central role in propagating fibrogenic signaling, establishing myofibroblasts as the major activated fibrogenic cell population in both wound repair and pathological stromal remodeling [42]. GSEA analysis further revealed that aCAF‐related genes were enriched in pathways regulating oxidative damage response, cancer‐related signaling, and the p53 pathway, with functional enrichment in chemical carcinogenesis, P450‐linked metabolism, and proinflammatory mediators, collectively highlighting their tumor‐promoting and immunosuppressive effects.

Our findings align with previous reports demonstrating that CAF risk signatures predict poor survival and reduced CD8+ T‐cell infiltration in CRC [3], and that FAP‐positive CAFs suppress CD8+ T‐cell‐mediated immunity, leading to worse clinical outcomes [43]. While most CAFs promote immunosuppression [44], certain subsets may paradoxically enhance immune activation under specific conditions [45]. Our classification of aCAFs into sCAFs and nsCAFs based on SASP expression revealed that sCAFs were linked to a poorer prognosis, challenging the conventional view that senescence exclusively suppresses tumor growth [8, 46]. This observation underscores the complexity of CAF biology and the need for refined classification systems.

Given the functional heterogeneity of CAFs and their diverse clinicopathological implications [47], detailed subclassification is essential for comprehensive understanding of their roles in tumor progression [48]. Our study addresses this need by integrating histological and molecular assessments to define aCAF subpopulations that exhibit molecular characteristics of myCAFs. By demonstrating that machine learning models incorporating aCAF status improve survival prediction, our findings provide a framework for incorporating morphologically defined CAF subtypes into prognostic algorithms and potentially therapeutic stratification in CRC.

This study has several limitations. First, the retrospective design may introduce confounding variables and selection bias, limiting generalizability. While the historical cohort minimized confounding from evolving therapies, this may limit applicability to contemporary treatment settings. Second, while aCAF identification was performed independently by three pathologists with substantial inter‐observer agreement, the assessment was based solely on morphological criteria using H&E‐stained sections without additional immunohistochemical or molecular validation. Third, functional validation of aCAF‐mediated immune suppression through experimental studies was not undertaken, leaving the observed association with reduced CD8^+^ T‐cell infiltration largely correlative. Fourth, the prognostic improvement from incorporating aCAFs into machine learning models was modest, and formal statistical comparison between models was not performed.

In summary, we explored the clinicopathological significance of aCAFs in CRC, focusing on their unique histopathological characteristics among various CAF subtypes previously described in the literature. We analyzed their impact on survival by elucidating various mechanisms through bioinformatics and developing predictive survival models using machine learning approaches. Notably, patients with aCAFs exhibiting SASP characteristics demonstrated significantly poorer survival outcomes compared to those without these features. The identification of aCAFs as a potential prognostic biomarker offers promising implications for personalized therapeutic strategies, including refined approaches to immunotherapy and targeted molecular interventions. Further experimental validation and prospective clinical studies are necessary to translate these findings into meaningful clinical applications.

## Author contributions statement

Conception and design: K‐WM, Y‐KN. Data acquisition: O‐ZK, BKS, JKJ, SWL, HSM, HSK, MJK, SHL. Data analysis/interpretation: K‐WM, Y‐KN. Writing – original draft: K‐WM, MS, O‐ZK. Supervision and critical review of the manuscript: K‐WM, Y‐KN. Final approval of submission: all.

## Supporting information


**Figure S1.** Schematic representation of the study design and workflow
**Table S1.** Interobserver assessment of aCAFs presence among three pathologists in our cohort
**Table S2.** Interobserver assessment of aCAFs presence among three pathologists in the TCGA dataset
**Table S3.** Correlation between clinicopathological parameters and activated cancer‐associated fibroblasts in the TCGA dataset
**Table S4.** Disease‐specific survival and overall survival analyses according to activated cancer‐associated fibroblasts in the TCGA dataset

## Data Availability

Public data used in this work can be acquired from the TCGA Research Network portal (https://gdc.cancer.gov/about-data/publications/pancanatlas). The raw experimental data and analysis codes supporting the conclusions of this article are available upon request from the corresponding author.
