# Supplementary material for: Cancer‐associated fibroblasts are associated with CD8+ T cell depletion and poor prognosis in colorectal adenocarcinoma: a multi‐omics and machine learning analysis
Source: J Pathol Clin Res. 2026 Feb 22;12(2):e70076. doi: 10.1002/2056-4538.70076 (PMC12928035; doi:10.1002/2056-4538.70076)
Supplement: Supplementary file 1 — Figure S1. Schematic representation of the study design and workflow Table S1. Interobserver assessment of aCAFs presence among three pathologists in our cohort Table S2. Interobserver assessment of aCAFs presence among three pathologists in the TCGA dataset Table S3. Correlation between clinicopathological parameters and activated cancer‐associated fibroblasts in the TCGA dataset Table S4. Disease‐specific survival and overall survival analyses according to activated cancer‐associated fibroblasts in the TCGA dataset [file CJP2-12-e70076-s001.pdf]

# Cancer-associated fibroblasts are associated with CD8+ T cell depletion and poor prognosis in colorectal adenocarcinoma: a multi-omics and machine learning analysis

M Shim, O-Z Kim *et al*. *J Pathol Clin Res* https://doi.org/10.1002/2056-4538.70076

**Supplementary Figure S1**
**Supplementary Tables S1–S4**

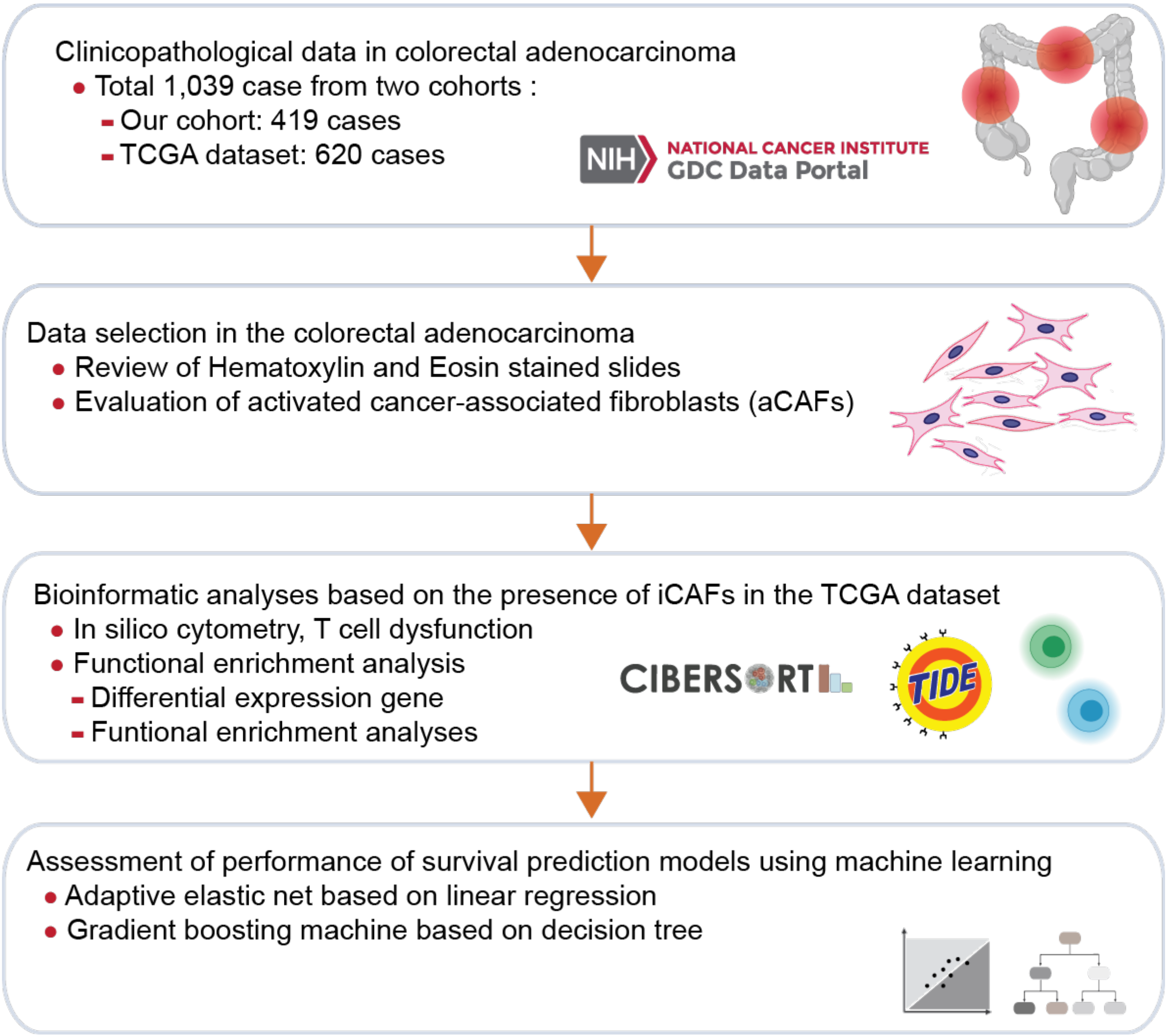


**Figure S1.** Schematic representation of the study design and workflow (Created with BioRender.com).

**Table S1**. Interobserver assessment of aCAFs presence among three pathologists in our cohort (404 cases)

| Cases | Observer 1 | Observer 2 | Observer 3 |
|---|---|---|---|
| 1 | present | present | present |
| 2 | present | present | present |
| 3 | not identified | not identified | not identified |
| 4 | present | present | present |
| 5 | not identified | not identified | not identified |
| 6 | not identified | not identified | not identified |
| 7 | not identified | not identified | not identified |
| 8 | present | present | present |
| 9 | not identified | not identified | not identified |
| 10 | not identified | not identified | not identified |
| 11 | not identified | not identified | not identified |
| 12 | not identified | not identified | not identified |
| 13 | not identified | not identified | not identified |
| 14 | present | present | present |
| 15 | present | present | not identified |
| 16 | not identified | not identified | present |
| 17 | present | present | present |
| 18 | not identified | present | not identified |
| 19 | not identified | not identified | not identified |
| 20 | not identified | not identified | not identified |
| 21 | not identified | not identified | not identified |
| 22 | not identified | not identified | not identified |
| 23 | not identified | not identified | not identified |
| 24 | not identified | not identified | not identified |
| 25 | not identified | not identified | not identified |
| 26 | not identified | not identified | not identified |
| 27 | not identified | not identified | present |
| 28 | not identified | not identified | not identified |
| 29 | not identified | not identified | not identified |
| 30 | not identified | not identified | not identified |
| 31 | not identified | present | not identified |
| 32 | not identified | not identified | not identified |
| 33 | present | present | present |
| 34 | present | not identified | present |
| 35 | not identified | not identified | not identified |
| 36 | not identified | not identified | not identified |
| 37 | not identified | not identified | not identified |
| 38 | not identified | not identified | not identified |
| 39 | not identified | not identified | not identified |
| 40 | not identified | not identified | not identified |
| 41 | not identified | not identified | not identified |
| 42 | not identified | not identified | not identified |
| 43 | not identified | not identified | not identified |
| 44 | not identified | not identified | not identified |
| 45 | not identified | not identified | not identified |

| | | | |
|---|---|---|---|
| 46 | not identified | not identified | not identified |
| 47 | present | present | present |
| 48 | present | present | present |
| 49 | present | present | present |
| 50 | not identified | not identified | not identified |
| 51 | not identified | not identified | not identified |
| 52 | not identified | not identified | present |
| 53 | present | present | present |
| 54 | not identified | not identified | not identified |
| 55 | present | present | present |
| 56 | not identified | not identified | not identified |
| 57 | not identified | not identified | not identified |
| 58 | present | present | present |
| 59 | not identified | not identified | not identified |
| 60 | not identified | not identified | not identified |
| 61 | not identified | not identified | not identified |
| 62 | present | present | not identified |
| 63 | present | present | present |
| 64 | present | present | present |
| 65 | not identified | not identified | not identified |
| 66 | not identified | not identified | not identified |
| 67 | not identified | present | not identified |
| 68 | not identified | not identified | present |
| 69 | not identified | not identified | not identified |
| 70 | not identified | not identified | not identified |
| 71 | not identified | not identified | not identified |
| 72 | not identified | present | not identified |
| 73 | not identified | not identified | not identified |
| 74 | present | present | present |
| 75 | not identified | not identified | not identified |
| 76 | not identified | not identified | not identified |
| 77 | not identified | not identified | not identified |
| 78 | not identified | not identified | not identified |
| 79 | present | present | present |
| 80 | not identified | not identified | not identified |
| 81 | not identified | not identified | not identified |
| 82 | not identified | not identified | not identified |
| 83 | not identified | not identified | not identified |
| 84 | not identified | not identified | not identified |
| 85 | not identified | not identified | not identified |
| 86 | not identified | not identified | not identified |
| 87 | not identified | not identified | not identified |
| 88 | not identified | not identified | not identified |
| 89 | not identified | not identified | not identified |
| 90 | not identified | not identified | not identified |
| 91 | not identified | not identified | not identified |
| 92 | not identified | not identified | not identified |
| 93 | not identified | not identified | not identified |

| | | | |
|---|---|---|---|
| 94 | not identified | not identified | not identified |
| 95 | not identified | not identified | not identified |
| 96 | not identified | not identified | not identified |
| 97 | not identified | not identified | not identified |
| 98 | not identified | not identified | not identified |
| 99 | present | present | present |
| 100 | not identified | present | not identified |
| 101 | present | present | present |
| 102 | not identified | not identified | not identified |
| 103 | not identified | not identified | present |
| 104 | not identified | not identified | not identified |
| 105 | not identified | not identified | not identified |
| 106 | not identified | not identified | not identified |
| 107 | not identified | not identified | not identified |
| 108 | not identified | not identified | not identified |
| 109 | not identified | not identified | not identified |
| 110 | not identified | present | not identified |
| 111 | present | present | not identified |
| 112 | present | present | present |
| 113 | not identified | not identified | not identified |
| 114 | not identified | not identified | not identified |
| 115 | not identified | not identified | not identified |
| 116 | present | present | not identified |
| 117 | present | present | present |
| 118 | not identified | not identified | not identified |
| 119 | not identified | not identified | not identified |
| 120 | not identified | not identified | not identified |
| 121 | not identified | not identified | not identified |
| 122 | not identified | not identified | not identified |
| 123 | not identified | not identified | not identified |
| 124 | not identified | not identified | not identified |
| 125 | present | present | present |
| 126 | not identified | not identified | not identified |
| 127 | not identified | not identified | present |
| 128 | not identified | not identified | not identified |
| 129 | not identified | not identified | not identified |
| 130 | not identified | not identified | not identified |
| 131 | present | present | present |
| 132 | present | present | present |
| 133 | not identified | not identified | not identified |
| 134 | present | present | present |
| 135 | present | present | present |
| 136 | not identified | not identified | not identified |
| 137 | not identified | not identified | not identified |
| 138 | not identified | not identified | not identified |
| 139 | not identified | not identified | present |
| 140 | not identified | not identified | not identified |
| 141 | not identified | not identified | not identified |

| | | | |
|---|---|---|---|
| 142 | not identified | not identified | not identified |
| 143 | not identified | not identified | not identified |
| 144 | not identified | not identified | not identified |
| 145 | not identified | not identified | not identified |
| 146 | not identified | not identified | not identified |
| 147 | not identified | not identified | not identified |
| 148 | not identified | not identified | not identified |
| 149 | not identified | not identified | not identified |
| 150 | not identified | not identified | not identified |
| 151 | not identified | not identified | not identified |
| 152 | present | present | present |
| 153 | not identified | not identified | not identified |
| 154 | not identified | not identified | not identified |
| 155 | not identified | not identified | not identified |
| 156 | not identified | not identified | not identified |
| 157 | not identified | not identified | not identified |
| 158 | not identified | not identified | not identified |
| 159 | present | present | present |
| 160 | not identified | not identified | not identified |
| 161 | present | present | present |
| 162 | not identified | not identified | not identified |
| 163 | present | present | present |
| 164 | not identified | not identified | not identified |
| 165 | present | present | present |
| 166 | present | present | present |
| 167 | not identified | not identified | not identified |
| 168 | not identified | not identified | not identified |
| 169 | not identified | not identified | not identified |
| 170 | present | present | present |
| 171 | not identified | not identified | not identified |
| 172 | present | present | present |
| 173 | present | present | present |
| 174 | present | present | present |
| 175 | present | present | present |
| 176 | present | present | present |
| 177 | not identified | not identified | not identified |
| 178 | present | present | present |
| 179 | present | present | not identified |
| 180 | not identified | not identified | not identified |
| 181 | not identified | not identified | not identified |
| 182 | present | present | present |
| 183 | not identified | not identified | not identified |
| 184 | not identified | not identified | not identified |
| 185 | present | present | present |
| 186 | not identified | present | not identified |
| 187 | not identified | not identified | not identified |
| 188 | not identified | not identified | not identified |
| 189 | not identified | not identified | not identified |

| | | | |
|---|---|---|---|
| 190 | present | present | not identified |
| 191 | not identified | not identified | not identified |
| 192 | not identified | not identified | not identified |
| 193 | not identified | not identified | not identified |
| 194 | not identified | not identified | not identified |
| 195 | not identified | not identified | not identified |
| 196 | not identified | not identified | not identified |
| 197 | present | present | not identified |
| 198 | not identified | not identified | not identified |
| 199 | not identified | not identified | not identified |
| 200 | present | present | present |
| 201 | not identified | not identified | not identified |
| 202 | not identified | not identified | not identified |
| 203 | present | present | present |
| 204 | not identified | not identified | present |
| 205 | not identified | not identified | not identified |
| 206 | not identified | not identified | not identified |
| 207 | not identified | not identified | not identified |
| 208 | not identified | not identified | not identified |
| 209 | not identified | not identified | not identified |
| 210 | present | present | present |
| 211 | not identified | not identified | not identified |
| 212 | not identified | not identified | not identified |
| 213 | not identified | not identified | not identified |
| 214 | not identified | not identified | not identified |
| 215 | not identified | not identified | not identified |
| 216 | not identified | not identified | not identified |
| 217 | present | present | present |
| 218 | not identified | not identified | present |
| 219 | not identified | not identified | not identified |
| 220 | present | present | present |
| 221 | present | present | present |
| 222 | not identified | not identified | not identified |
| 223 | not identified | not identified | not identified |
| 224 | not identified | not identified | not identified |
| 225 | not identified | not identified | not identified |
| 226 | not identified | not identified | not identified |
| 227 | not identified | not identified | not identified |
| 228 | not identified | not identified | not identified |
| 229 | not identified | not identified | not identified |
| 230 | not identified | not identified | not identified |
| 231 | not identified | not identified | not identified |
| 232 | present | present | present |
| 233 | not identified | not identified | not identified |
| 234 | not identified | not identified | not identified |
| 235 | not identified | not identified | not identified |
| 236 | not identified | not identified | not identified |
| 237 | present | present | present |

| | | | |
|---|---|---|---|
| 238 | not identified | not identified | not identified |
| 239 | not identified | not identified | not identified |
| 240 | not identified | not identified | not identified |
| 241 | not identified | not identified | not identified |
| 242 | present | present | present |
| 243 | not identified | not identified | not identified |
| 244 | not identified | not identified | not identified |
| 245 | not identified | not identified | not identified |
| 246 | not identified | present | not identified |
| 247 | not identified | not identified | not identified |
| 248 | not identified | not identified | not identified |
| 249 | not identified | not identified | present |
| 250 | present | present | present |
| 251 | not identified | present | not identified |
| 252 | not identified | not identified | present |
| 253 | not identified | not identified | not identified |
| 254 | not identified | not identified | not identified |
| 255 | not identified | not identified | not identified |
| 256 | not identified | not identified | not identified |
| 257 | not identified | not identified | not identified |
| 258 | not identified | not identified | not identified |
| 259 | not identified | not identified | not identified |
| 260 | present | present | present |
| 261 | not identified | not identified | not identified |
| 262 | present | present | present |
| 263 | present | present | present |
| 264 | not identified | present | not identified |
| 265 | not identified | not identified | not identified |
| 266 | present | present | present |
| 267 | present | present | present |
| 268 | present | present | present |
| 269 | not identified | not identified | not identified |
| 270 | not identified | not identified | not identified |
| 271 | not identified | not identified | present |
| 272 | present | present | present |
| 273 | not identified | not identified | not identified |
| 274 | present | present | not identified |
| 275 | not identified | not identified | not identified |
| 276 | not identified | not identified | not identified |
| 277 | not identified | not identified | not identified |
| 278 | not identified | not identified | not identified |
| 279 | present | present | present |
| 280 | not identified | not identified | not identified |
| 281 | not identified | not identified | not identified |
| 282 | present | present | present |
| 283 | not identified | not identified | not identified |
| 284 | present | present | present |
| 285 | present | present | present |

| | | | |
|---|---|---|---|
| 286 | not identified | not identified | not identified |
| 287 | not identified | not identified | not identified |
| 288 | not identified | not identified | not identified |
| 289 | not identified | present | not identified |
| 290 | not identified | not identified | not identified |
| 291 | present | present | present |
| 292 | not identified | not identified | not identified |
| 293 | not identified | not identified | not identified |
| 294 | not identified | not identified | not identified |
| 295 | not identified | not identified | not identified |
| 296 | not identified | not identified | not identified |
| 297 | not identified | not identified | not identified |
| 298 | not identified | not identified | not identified |
| 299 | present | not identified | present |
| 300 | present | present | present |
| 301 | not identified | not identified | not identified |
| 302 | not identified | not identified | not identified |
| 303 | not identified | present | not identified |
| 304 | present | present | present |
| 305 | present | present | present |
| 306 | not identified | not identified | not identified |
| 307 | present | present | present |
| 308 | not identified | not identified | not identified |
| 309 | not identified | not identified | not identified |
| 310 | not identified | not identified | not identified |
| 311 | not identified | not identified | not identified |
| 312 | present | present | present |
| 313 | not identified | not identified | present |
| 314 | not identified | present | not identified |
| 315 | not identified | not identified | not identified |
| 316 | not identified | not identified | not identified |
| 317 | not identified | not identified | present |
| 318 | not identified | not identified | not identified |
| 319 | present | present | present |
| 320 | not identified | not identified | not identified |
| 321 | not identified | not identified | not identified |
| 322 | not identified | not identified | not identified |
| 323 | not identified | not identified | not identified |
| 324 | not identified | present | not identified |
| 325 | present | present | present |
| 326 | not identified | not identified | not identified |
| 327 | not identified | not identified | not identified |
| 328 | not identified | not identified | not identified |
| 329 | not identified | not identified | not identified |
| 330 | not identified | not identified | not identified |
| 331 | not identified | not identified | not identified |
| 332 | not identified | not identified | not identified |
| 333 | not identified | not identified | not identified |

| | | | |
|---|---|---|---|
| 334 | present | present | present |
| 335 | not identified | not identified | not identified |
| 336 | not identified | not identified | present |
| 337 | not identified | not identified | not identified |
| 338 | not identified | not identified | not identified |
| 339 | not identified | not identified | not identified |
| 340 | present | present | present |
| 341 | not identified | not identified | not identified |
| 342 | not identified | not identified | present |
| 343 | not identified | not identified | not identified |
| 344 | not identified | not identified | not identified |
| 345 | not identified | not identified | not identified |
| 346 | present | present | present |
| 347 | not identified | not identified | not identified |
| 348 | not identified | not identified | not identified |
| 349 | not identified | not identified | not identified |
| 350 | not identified | not identified | not identified |
| 351 | not identified | not identified | not identified |
| 352 | present | present | not identified |
| 353 | not identified | not identified | not identified |
| 354 | not identified | not identified | not identified |
| 355 | present | present | present |
| 356 | not identified | not identified | not identified |
| 357 | not identified | not identified | not identified |
| 358 | present | present | present |
| 359 | not identified | present | present |
| 360 | not identified | not identified | not identified |
| 361 | not identified | not identified | not identified |
| 362 | not identified | not identified | present |
| 363 | not identified | not identified | not identified |
| 364 | not identified | not identified | not identified |
| 365 | present | not identified | present |
| 366 | not identified | not identified | not identified |
| 367 | not identified | not identified | present |
| 368 | present | present | not identified |
| 369 | not identified | present | not identified |
| 370 | not identified | not identified | not identified |
| 371 | not identified | not identified | not identified |
| 372 | present | present | present |
| 373 | present | not identified | present |
| 374 | not identified | not identified | not identified |
| 375 | not identified | not identified | not identified |
| 376 | not identified | not identified | not identified |
| 377 | present | present | present |
| 378 | not identified | not identified | present |
| 379 | not identified | not identified | not identified |
| 380 | not identified | not identified | not identified |
| 381 | present | present | present |

| | | | |
|---|---|---|---|
| 382 | not identified | not identified | present |
| 383 | not identified | not identified | not identified |
| 384 | not identified | not identified | not identified |
| 385 | not identified | not identified | not identified |
| 386 | not identified | not identified | not identified |
| 387 | not identified | not identified | not identified |
| 388 | not identified | present | not identified |
| 389 | present | present | present |
| 390 | not identified | not identified | not identified |
| 391 | present | present | present |
| 392 | not identified | not identified | not identified |
| 393 | not identified | not identified | not identified |
| 394 | not identified | not identified | not identified |
| 395 | present | present | present |
| 396 | not identified | not identified | not identified |
| 397 | present | present | not identified |
| 398 | not identified | not identified | not identified |
| 399 | present | not identified | present |
| 400 | present | present | present |
| 401 | present | present | present |
| 402 | not identified | not identified | not identified |
| 403 | not identified | not identified | not identified |
| 404 | not identified | not identified | not identified |

**Table S2**. Interobserver assessment of aCAFs presence among three pathologists in the TCGA dataset

| Cases | Observer 1 | Observer 2 | Observer 3 |
|---|---|---|---|
| 1 | present | present | present |
| 2 | not identified | not identified | not identified |
| 3 | not identified | not identified | not identified |
| 4 | not identified | not identified | present |
| 5 | present | present | not identified |
| 6 | present | present | present |
| 7 | not identified | not identified | present |
| 8 | not identified | not identified | not identified |
| 9 | not identified | not identified | not identified |
| 10 | present | not identified | present |
| 11 | not identified | present | present |
| 12 | present | present | present |
| 13 | not identified | not identified | not identified |
| 14 | not identified | not identified | not identified |
| 15 | not identified | not identified | not identified |
| 16 | present | present | not identified |
| 17 | present | present | present |
| 18 | not identified | present | present |
| 19 | present | present | present |
| 20 | not identified | not identified | not identified |
| 21 | present | present | present |
| 22 | present | present | not identified |
| 23 | present | present | present |
| 24 | not identified | not identified | not identified |
| 25 | not identified | not identified | not identified |
| 26 | not identified | not identified | not identified |
| 27 | present | present | not identified |
| 28 | not identified | not identified | not identified |
| 29 | present | present | present |
| 30 | present | present | present |
| 31 | present | present | present |
| 32 | not identified | not identified | not identified |
| 33 | not identified | not identified | not identified |
| 34 | not identified | not identified | not identified |
| 35 | not identified | not identified | not identified |
| 36 | present | not identified | present |
| 37 | present | present | present |
| 38 | not identified | not identified | not identified |
| 39 | not identified | not identified | not identified |
| 40 | not identified | not identified | not identified |
| 41 | not identified | not identified | not identified |
| 42 | not identified | not identified | not identified |
| 43 | present | present | present |
| 44 | present | present | present |
| 45 | not identified | not identified | not identified |

| | | | |
|---|---|---|---|
| 46 | present | not identified | present |
| 47 | not identified | not identified | not identified |
| 48 | present | present | present |
| 49 | present | present | present |
| 50 | not identified | not identified | not identified |
| 51 | not identified | not identified | not identified |
| 52 | not identified | not identified | not identified |
| 53 | not identified | present | not identified |
| 54 | not identified | not identified | present |
| 55 | present | present | present |
| 56 | not identified | not identified | not identified |
| 57 | not identified | not identified | not identified |
| 58 | not identified | not identified | present |
| 59 | not identified | not identified | present |
| 60 | not identified | not identified | not identified |
| 61 | not identified | not identified | not identified |
| 62 | not identified | not identified | not identified |
| 63 | not identified | not identified | not identified |
| 64 | not identified | not identified | not identified |
| 65 | not identified | not identified | not identified |
| 66 | not identified | not identified | not identified |
| 67 | not identified | not identified | not identified |
| 68 | not identified | not identified | not identified |
| 69 | not identified | not identified | not identified |
| 70 | not identified | not identified | not identified |
| 71 | present | present | present |
| 72 | not identified | not identified | not identified |
| 73 | not identified | not identified | not identified |
| 74 | not identified | not identified | not identified |
| 75 | not identified | not identified | not identified |
| 76 | not identified | not identified | not identified |
| 77 | not identified | not identified | not identified |
| 78 | not identified | not identified | not identified |
| 79 | not identified | not identified | not identified |
| 80 | present | present | present |
| 81 | not identified | not identified | not identified |
| 82 | not identified | not identified | not identified |
| 83 | not identified | not identified | not identified |
| 84 | not identified | not identified | not identified |
| 85 | present | not identified | not identified |
| 86 | not identified | not identified | not identified |
| 87 | not identified | not identified | not identified |
| 88 | not identified | not identified | not identified |
| 89 | not identified | not identified | not identified |
| 90 | not identified | not identified | not identified |
| 91 | not identified | present | not identified |
| 92 | not identified | not identified | not identified |
| 93 | not identified | not identified | not identified |

| | | | |
|---|---|---|---|
| 94 | not identified | not identified | not identified |
| 95 | not identified | not identified | not identified |
| 96 | not identified | not identified | not identified |
| 97 | not identified | not identified | not identified |
| 98 | not identified | not identified | not identified |
| 99 | not identified | not identified | not identified |
| 100 | not identified | not identified | not identified |
| 101 | not identified | present | present |
| 102 | not identified | not identified | not identified |
| 103 | not identified | not identified | not identified |
| 104 | not identified | not identified | not identified |
| 105 | not identified | not identified | not identified |
| 106 | present | not identified | not identified |
| 107 | not identified | present | not identified |
| 108 | not identified | not identified | not identified |
| 109 | not identified | not identified | not identified |
| 110 | not identified | not identified | not identified |
| 111 | not identified | not identified | not identified |
| 112 | not identified | present | not identified |
| 113 | not identified | not identified | not identified |
| 114 | not identified | not identified | present |
| 115 | not identified | not identified | not identified |
| 116 | present | present | present |
| 117 | not identified | not identified | not identified |
| 118 | not identified | not identified | not identified |
| 119 | not identified | not identified | not identified |
| 120 | not identified | not identified | not identified |
| 121 | not identified | not identified | not identified |
| 122 | not identified | not identified | not identified |
| 123 | not identified | not identified | not identified |
| 124 | not identified | not identified | not identified |
| 125 | not identified | not identified | not identified |
| 126 | not identified | not identified | not identified |
| 127 | present | present | present |
| 128 | not identified | not identified | not identified |
| 129 | not identified | not identified | not identified |
| 130 | not identified | not identified | not identified |
| 131 | present | present | present |
| 132 | not identified | not identified | not identified |
| 133 | present | present | present |
| 134 | not identified | not identified | present |
| 135 | not identified | not identified | not identified |
| 136 | not identified | not identified | present |
| 137 | not identified | not identified | not identified |
| 138 | not identified | not identified | not identified |
| 139 | not identified | not identified | not identified |
| 140 | not identified | not identified | not identified |
| 141 | present | present | present |

| | | | |
|---|---|---|---|
| 142 | not identified | not identified | not identified |
| 143 | not identified | not identified | not identified |
| 144 | not identified | not identified | not identified |
| 145 | not identified | not identified | not identified |
| 146 | not identified | not identified | not identified |
| 147 | not identified | not identified | not identified |
| 148 | present | present | present |
| 149 | present | present | not identified |
| 150 | not identified | not identified | not identified |
| 151 | not identified | not identified | not identified |
| 152 | present | present | not identified |
| 153 | not identified | not identified | not identified |
| 154 | present | present | present |
| 155 | present | present | present |
| 156 | present | present | present |
| 157 | not identified | not identified | not identified |
| 158 | not identified | not identified | not identified |
| 159 | not identified | not identified | not identified |
| 160 | present | present | present |
| 161 | present | present | present |
| 162 | not identified | not identified | not identified |
| 163 | not identified | not identified | not identified |
| 164 | present | present | present |
| 165 | not identified | not identified | not identified |
| 166 | not identified | not identified | not identified |
| 167 | present | present | present |
| 168 | present | present | present |
| 169 | present | present | present |
| 170 | not identified | not identified | not identified |
| 171 | not identified | not identified | not identified |
| 172 | not identified | not identified | not identified |
| 173 | not identified | not identified | not identified |
| 174 | not identified | not identified | not identified |
| 175 | not identified | not identified | not identified |
| 176 | not identified | not identified | not identified |
| 177 | not identified | not identified | not identified |
| 178 | not identified | not identified | not identified |
| 179 | not identified | not identified | not identified |
| 180 | not identified | not identified | not identified |
| 181 | not identified | not identified | not identified |
| 182 | present | present | present |
| 183 | not identified | not identified | not identified |
| 184 | present | present | present |
| 185 | not identified | not identified | not identified |
| 186 | not identified | not identified | not identified |
| 187 | not identified | not identified | not identified |
| 188 | not identified | not identified | not identified |
| 189 | not identified | not identified | not identified |

| | | | |
|---|---|---|---|
| 190 | not identified | not identified | not identified |
| 191 | not identified | not identified | not identified |
| 192 | not identified | not identified | not identified |
| 193 | not identified | not identified | not identified |
| 194 | not identified | not identified | not identified |
| 195 | not identified | not identified | not identified |
| 196 | not identified | not identified | not identified |
| 197 | not identified | not identified | not identified |
| 198 | not identified | not identified | not identified |
| 199 | not identified | not identified | not identified |
| 200 | not identified | not identified | not identified |
| 201 | not identified | not identified | not identified |
| 202 | not identified | not identified | not identified |
| 203 | not identified | not identified | not identified |
| 204 | not identified | not identified | not identified |
| 205 | not identified | not identified | not identified |
| 206 | not identified | not identified | not identified |
| 207 | not identified | not identified | not identified |
| 208 | not identified | not identified | not identified |
| 209 | not identified | not identified | not identified |
| 210 | not identified | not identified | not identified |
| 211 | not identified | not identified | not identified |
| 212 | not identified | not identified | not identified |
| 213 | not identified | not identified | not identified |
| 214 | present | present | present |
| 215 | present | present | present |
| 216 | not identified | not identified | not identified |
| 217 | not identified | not identified | not identified |
| 218 | present | present | not identified |
| 219 | not identified | not identified | not identified |
| 220 | not identified | not identified | not identified |
| 221 | not identified | not identified | not identified |
| 222 | not identified | not identified | not identified |
| 223 | not identified | not identified | not identified |
| 224 | not identified | not identified | not identified |
| 225 | present | present | present |
| 226 | not identified | not identified | not identified |
| 227 | not identified | not identified | not identified |
| 228 | not identified | not identified | not identified |
| 229 | not identified | not identified | not identified |
| 230 | present | present | present |
| 231 | not identified | not identified | not identified |
| 232 | not identified | not identified | not identified |
| 233 | not identified | not identified | not identified |
| 234 | not identified | not identified | not identified |
| 235 | not identified | not identified | not identified |
| 236 | not identified | not identified | not identified |
| 237 | present | present | present |

| | | | |
|---|---|---|---|
| 238 | not identified | not identified | not identified |
| 239 | not identified | not identified | not identified |
| 240 | present | not identified | present |
| 241 | not identified | present | not identified |
| 242 | not identified | not identified | not identified |
| 243 | not identified | not identified | not identified |
| 244 | not identified | not identified | not identified |
| 245 | not identified | not identified | not identified |
| 246 | not identified | not identified | not identified |
| 247 | present | present | present |
| 248 | present | present | present |
| 249 | not identified | not identified | not identified |
| 250 | present | present | present |
| 251 | not identified | not identified | not identified |
| 252 | not identified | not identified | not identified |
| 253 | not identified | not identified | not identified |
| 254 | present | present | present |
| 255 | present | present | present |
| 256 | present | present | present |
| 257 | not identified | not identified | not identified |
| 258 | not identified | not identified | not identified |
| 259 | present | present | present |
| 260 | present | present | present |
| 261 | not identified | not identified | not identified |
| 262 | not identified | not identified | not identified |
| 263 | not identified | not identified | not identified |
| 264 | present | present | not identified |
| 265 | present | present | present |
| 266 | not identified | not identified | not identified |
| 267 | present | present | not identified |
| 268 | present | present | present |
| 269 | present | present | present |
| 270 | not identified | not identified | not identified |
| 271 | present | present | present |
| 272 | present | present | present |
| 273 | not identified | not identified | not identified |
| 274 | not identified | not identified | not identified |
| 275 | not identified | not identified | not identified |
| 276 | present | present | present |
| 277 | not identified | not identified | not identified |
| 278 | not identified | not identified | not identified |
| 279 | not identified | not identified | not identified |
| 280 | present | present | present |
| 281 | present | present | present |
| 282 | not identified | not identified | not identified |
| 283 | present | present | present |
| 284 | not identified | not identified | not identified |
| 285 | not identified | not identified | not identified |

| | | | |
|---|---|---|---|
| 286 | not identified | not identified | not identified |
| 287 | not identified | not identified | not identified |
| 288 | not identified | not identified | not identified |
| 289 | not identified | not identified | not identified |
| 290 | present | present | not identified |
| 291 | not identified | not identified | not identified |
| 292 | not identified | not identified | not identified |
| 293 | not identified | not identified | not identified |
| 294 | not identified | not identified | not identified |
| 295 | not identified | not identified | not identified |
| 296 | not identified | not identified | not identified |
| 297 | not identified | not identified | not identified |
| 298 | present | present | present |
| 299 | not identified | not identified | not identified |
| 300 | not identified | not identified | not identified |
| 301 | present | present | present |
| 302 | not identified | not identified | not identified |
| 303 | not identified | not identified | not identified |
| 304 | not identified | not identified | not identified |
| 305 | present | present | present |
| 306 | not identified | not identified | not identified |
| 307 | present | present | present |
| 308 | present | present | present |
| 309 | present | not identified | present |
| 310 | not identified | not identified | not identified |
| 311 | not identified | not identified | not identified |
| 312 | not identified | not identified | not identified |
| 313 | not identified | not identified | not identified |
| 314 | not identified | not identified | not identified |
| 315 | not identified | not identified | not identified |
| 316 | not identified | not identified | not identified |
| 317 | not identified | not identified | not identified |
| 318 | not identified | not identified | not identified |
| 319 | not identified | not identified | not identified |
| 320 | not identified | not identified | not identified |
| 321 | not identified | not identified | not identified |
| 322 | not identified | not identified | not identified |
| 323 | not identified | not identified | not identified |
| 324 | not identified | not identified | not identified |
| 325 | not identified | not identified | not identified |
| 326 | present | present | present |
| 327 | not identified | not identified | not identified |
| 328 | not identified | not identified | not identified |
| 329 | not identified | not identified | not identified |
| 330 | present | present | not identified |
| 331 | not identified | not identified | not identified |
| 332 | present | present | present |
| 333 | present | present | present |

| | | | |
|---|---|---|---|
| 334 | not identified | not identified | not identified |
| 335 | not identified | not identified | not identified |
| 336 | not identified | not identified | not identified |
| 337 | not identified | not identified | not identified |
| 338 | not identified | not identified | not identified |
| 339 | not identified | not identified | not identified |
| 340 | not identified | not identified | not identified |
| 341 | not identified | not identified | not identified |
| 342 | not identified | not identified | not identified |
| 343 | not identified | not identified | not identified |
| 344 | not identified | not identified | not identified |
| 345 | present | present | present |
| 346 | not identified | not identified | not identified |
| 347 | present | present | present |
| 348 | not identified | not identified | not identified |
| 349 | not identified | not identified | not identified |
| 350 | not identified | not identified | not identified |
| 351 | not identified | not identified | not identified |
| 352 | not identified | not identified | not identified |
| 353 | not identified | not identified | not identified |
| 354 | not identified | not identified | not identified |
| 355 | not identified | not identified | not identified |
| 356 | not identified | not identified | not identified |
| 357 | not identified | not identified | not identified |
| 358 | not identified | not identified | not identified |
| 359 | not identified | not identified | not identified |
| 360 | not identified | not identified | not identified |
| 361 | not identified | not identified | not identified |
| 362 | not identified | not identified | not identified |
| 363 | present | present | present |
| 364 | not identified | not identified | not identified |
| 365 | not identified | not identified | not identified |
| 366 | not identified | not identified | not identified |
| 367 | present | present | present |
| 368 | not identified | not identified | not identified |
| 369 | present | present | present |
| 370 | not identified | not identified | not identified |
| 371 | not identified | not identified | not identified |
| 372 | not identified | not identified | not identified |
| 373 | not identified | not identified | not identified |
| 374 | not identified | not identified | not identified |
| 375 | not identified | not identified | not identified |
| 376 | present | present | present |
| 377 | present | present | present |
| 378 | present | not identified | present |
| 379 | present | present | present |
| 380 | not identified | not identified | not identified |
| 381 | not identified | not identified | not identified |

| | | | |
|---|---|---|---|
| 382 | not identified | not identified | not identified |
| 383 | present | present | not identified |
| 384 | not identified | not identified | not identified |
| 385 | not identified | not identified | not identified |
| 386 | not identified | not identified | not identified |
| 387 | not identified | not identified | not identified |
| 388 | not identified | not identified | not identified |
| 389 | not identified | not identified | not identified |
| 390 | present | present | present |
| 391 | not identified | not identified | not identified |
| 392 | present | present | present |
| 393 | not identified | not identified | not identified |
| 394 | not identified | not identified | not identified |
| 395 | not identified | not identified | not identified |
| 396 | not identified | not identified | not identified |
| 397 | not identified | not identified | not identified |
| 398 | not identified | not identified | not identified |
| 399 | not identified | not identified | not identified |
| 400 | present | present | present |
| 401 | not identified | not identified | not identified |
| 402 | present | present | present |
| 403 | not identified | not identified | not identified |
| 404 | not identified | not identified | not identified |
| 405 | not identified | not identified | not identified |
| 406 | not identified | not identified | not identified |
| 407 | not identified | not identified | not identified |
| 408 | present | present | present |
| 409 | not identified | not identified | not identified |
| 410 | not identified | not identified | present |
| 411 | present | present | present |
| 412 | not identified | not identified | not identified |
| 413 | not identified | not identified | not identified |
| 414 | not identified | not identified | not identified |
| 415 | not identified | not identified | not identified |
| 416 | present | not identified | present |
| 417 | not identified | not identified | not identified |
| 418 | not identified | not identified | not identified |
| 419 | not identified | not identified | not identified |
| 420 | not identified | present | not identified |
| 421 | present | present | present |
| 422 | not identified | not identified | not identified |
| 423 | not identified | not identified | not identified |
| 424 | not identified | not identified | not identified |
| 425 | not identified | not identified | not identified |
| 426 | not identified | not identified | not identified |
| 427 | not identified | not identified | not identified |
| 428 | not identified | not identified | not identified |
| 429 | not identified | not identified | not identified |

| | | | |
|---|---|---|---|
| 430 | not identified | not identified | not identified |
| 431 | not identified | not identified | not identified |
| 432 | not identified | not identified | not identified |
| 433 | not identified | not identified | not identified |
| 434 | not identified | not identified | not identified |
| 435 | present | present | present |
| 436 | present | not identified | present |
| 437 | not identified | not identified | not identified |
| 438 | not identified | not identified | not identified |
| 439 | present | present | present |
| 440 | present | present | not identified |
| 441 | not identified | not identified | not identified |
| 442 | not identified | not identified | not identified |
| 443 | present | present | present |
| 444 | not identified | not identified | not identified |
| 445 | not identified | not identified | not identified |
| 446 | not identified | not identified | not identified |
| 447 | not identified | not identified | not identified |
| 448 | not identified | not identified | not identified |
| 449 | not identified | not identified | not identified |
| 450 | not identified | not identified | not identified |
| 451 | present | present | present |
| 452 | not identified | not identified | not identified |
| 453 | not identified | not identified | not identified |
| 454 | not identified | not identified | not identified |
| 455 | not identified | not identified | not identified |
| 456 | present | present | present |
| 457 | present | present | present |
| 458 | present | present | present |
| 459 | not identified | not identified | not identified |
| 460 | not identified | not identified | not identified |
| 461 | not identified | not identified | not identified |
| 462 | present | present | present |
| 463 | not identified | not identified | not identified |
| 464 | present | present | present |
| 465 | present | present | present |
| 466 | not identified | not identified | not identified |
| 467 | not identified | not identified | not identified |
| 468 | not identified | not identified | not identified |
| 469 | not identified | not identified | not identified |
| 470 | not identified | not identified | not identified |
| 471 | not identified | not identified | not identified |
| 472 | present | present | present |
| 473 | not identified | not identified | not identified |
| 474 | not identified | not identified | not identified |
| 475 | present | present | present |
| 476 | not identified | not identified | not identified |
| 477 | not identified | not identified | not identified |

| | | | |
|---|---|---|---|
| 478 | not identified | not identified | not identified |
| 479 | not identified | not identified | not identified |
| 480 | not identified | not identified | not identified |
| 481 | not identified | not identified | not identified |
| 482 | not identified | not identified | not identified |
| 483 | not identified | not identified | not identified |
| 484 | not identified | not identified | not identified |
| 485 | not identified | not identified | not identified |
| 486 | not identified | not identified | not identified |
| 487 | not identified | not identified | not identified |
| 488 | present | present | present |
| 489 | not identified | not identified | not identified |
| 490 | not identified | not identified | not identified |
| 491 | not identified | not identified | not identified |
| 492 | not identified | not identified | not identified |
| 493 | not identified | not identified | not identified |
| 494 | not identified | not identified | not identified |
| 495 | not identified | not identified | not identified |
| 496 | not identified | not identified | not identified |
| 497 | not identified | not identified | not identified |
| 498 | not identified | not identified | not identified |
| 499 | not identified | not identified | not identified |
| 500 | not identified | not identified | not identified |
| 501 | not identified | not identified | not identified |
| 502 | not identified | not identified | not identified |
| 503 | not identified | not identified | not identified |
| 504 | not identified | not identified | not identified |
| 505 | not identified | not identified | not identified |
| 506 | not identified | not identified | not identified |
| 507 | not identified | not identified | not identified |
| 508 | not identified | not identified | not identified |
| 509 | not identified | not identified | not identified |
| 510 | present | present | not identified |
| 511 | present | not identified | not identified |
| 512 | not identified | not identified | not identified |
| 513 | not identified | not identified | not identified |
| 514 | not identified | not identified | not identified |
| 515 | not identified | not identified | not identified |
| 516 | present | present | present |
| 517 | not identified | not identified | not identified |
| 518 | not identified | not identified | not identified |
| 519 | present | present | present |
| 520 | not identified | not identified | not identified |
| 521 | not identified | not identified | not identified |
| 522 | present | present | present |
| 523 | not identified | not identified | not identified |
| 524 | not identified | not identified | not identified |
| 525 | not identified | not identified | not identified |

| | | | |
|---|---|---|---|
| 526 | not identified | not identified | not identified |
| 527 | not identified | not identified | not identified |
| 528 | not identified | not identified | not identified |
| 529 | not identified | not identified | not identified |
| 530 | not identified | not identified | not identified |
| 531 | not identified | not identified | not identified |
| 532 | not identified | not identified | not identified |
| 533 | not identified | not identified | not identified |
| 534 | not identified | not identified | not identified |
| 535 | not identified | not identified | not identified |
| 536 | not identified | not identified | not identified |
| 537 | not identified | not identified | not identified |
| 538 | not identified | not identified | not identified |
| 539 | present | present | not identified |
| 540 | not identified | not identified | not identified |
| 541 | present | not identified | present |
| 542 | present | present | present |
| 543 | not identified | not identified | not identified |
| 544 | not identified | not identified | not identified |
| 545 | not identified | not identified | not identified |
| 546 | not identified | not identified | not identified |
| 547 | not identified | not identified | not identified |
| 548 | not identified | not identified | not identified |
| 549 | present | not identified | not identified |
| 550 | present | not identified | present |
| 551 | not identified | not identified | not identified |
| 552 | present | present | not identified |
| 553 | not identified | not identified | not identified |
| 554 | not identified | not identified | not identified |
| 555 | not identified | not identified | not identified |
| 556 | not identified | not identified | not identified |
| 557 | not identified | not identified | not identified |
| 558 | not identified | not identified | not identified |
| 559 | not identified | not identified | not identified |
| 560 | not identified | not identified | not identified |
| 561 | present | present | present |
| 562 | not identified | not identified | not identified |
| 563 | not identified | not identified | not identified |
| 564 | not identified | not identified | not identified |
| 565 | not identified | not identified | not identified |
| 566 | present | not identified | present |
| 567 | present | present | present |
| 568 | present | present | present |
| 569 | present | not identified | present |
| 570 | not identified | not identified | not identified |
| 571 | not identified | not identified | not identified |
| 572 | not identified | not identified | not identified |
| 573 | not identified | not identified | not identified |

| | | | |
|---|---|---|---|
| 574 | present | present | present |
| 575 | not identified | not identified | not identified |
| 576 | not identified | not identified | not identified |
| 577 | not identified | not identified | not identified |
| 578 | not identified | not identified | not identified |
| 579 | not identified | not identified | not identified |
| 580 | not identified | not identified | not identified |
| 581 | not identified | not identified | not identified |
| 582 | present | present | present |
| 583 | not identified | not identified | not identified |
| 584 | not identified | present | not identified |
| 585 | not identified | not identified | not identified |
| 586 | not identified | not identified | not identified |
| 587 | not identified | not identified | not identified |
| 588 | not identified | not identified | not identified |
| 589 | not identified | not identified | not identified |
| 590 | not identified | not identified | not identified |
| 591 | not identified | not identified | not identified |
| 592 | not identified | not identified | not identified |
| 593 | not identified | not identified | not identified |
| 594 | present | present | not identified |
| 595 | not identified | not identified | not identified |
| 596 | not identified | not identified | not identified |
| 597 | not identified | not identified | not identified |
| 598 | present | not identified | present |
| 599 | not identified | not identified | not identified |
| 600 | not identified | not identified | not identified |
| 601 | not identified | not identified | not identified |
| 602 | not identified | not identified | not identified |
| 603 | not identified | not identified | not identified |
| 604 | not identified | not identified | not identified |
| 605 | present | not identified | not identified |
| 606 | not identified | not identified | not identified |
| 607 | present | not identified | present |
| 608 | not identified | not identified | not identified |
| 609 | not identified | not identified | not identified |
| 610 | not identified | not identified | not identified |
| 611 | present | present | present |
| 612 | not identified | not identified | not identified |
| 613 | not identified | not identified | not identified |
| 614 | not identified | not identified | not identified |
| 615 | present | not identified | present |
| 616 | present | present | not identified |
| 617 | present | present | present |
| 618 | not identified | not identified | not identified |
| 619 | not identified | not identified | not identified |
| 620 | not identified | not identified | not identified |

**Table S3.** Correlation between clinicopathological parameters and activated cancer-associated fibroblasts in the TCGA dataset (617 cases)

| Parameter | Activated cancer-associated fibroblasts | | $p$-value |
|---|---|---|---|
| | Absence ($n$ = 478), $n$ (%) | Presence ($n$ = 142), $n$ (%) | $\chi^2$ |
| Age (years) | 66.3 ± 12.9 | 65.8 ± 12.3 | 0.679[a] |
| Tumor size (cm) | 5.1 ± 2.3 | 5.5 ± 2.1 | 0.138[a] |
| Sex | | | |
| Female | 227 (47.5) | 62 (43.7) | 0.48 |
| Male | 251 (52.5) | 80 (56.3) | |
| T stage | | | |
| 1 | 17 ( 3.6) | 3 ( 2.1) | **0.009[b]** |
| 2 | 91 (19.2) | 17 (12.0) | |
| 3 | 320 (67.4) | 100 (70.4) | |
| 4 | 47 ( 9.9) | 22 (15.5) | |
| N stage | | | |
| 0 | 298 (62.6) | 55 (39.0) | **<0.001[b]** |
| 1 | 103 (21.6) | 45 (31.9) | |
| 2 | 75 (15.8) | 41 (29.1) | |
| Location | | | |
| Cecum to sigmoid | 344 (72) | 108 (76.1) | 0.336 |
| Rectosigmoid to rectum | 134 (28) | 34 (23.9) | |
| Histological grade | | | |
| Well differentiated | 27 ( 5.6) | 6 ( 4.2) | **0.507[c]** |
| Moderately differentiated | 357 (74.7) | 108 (76.1) | |
| Poorly differentiated | 94 (19.7) | 28 (19.7) | |
| Lymphovascular invasion[d] | | | |
| Negative | 277 (58.4) | 63 (44.4) | **0.004** |
| Positive | 197 (41.6) | 79 (55.6) | |
| Perineural invasion[d] | | | |
| Negative | 137 (77.4) | 38 (60.3) | **0.014** |
| Positive | 40 (22.6) | 25 (39.7) | |
| Tumor necrosis | | | |
| Negative | 175 (36.6) | 25 (17.6) | **<0.001** |
| Positive | 301 (63.4) | 117 (82.4) | |

T or N stage, The 8th edition of the American Joint Committee on Cancer.

[a]Student's $t$-test.

[b]Linear-by-linear association.

[c]Well differentiated versus moderately differentiated and poorly differentiated.

[d]Some variables contained missing annotations.

**Table S4.** Disease-specific survival and overall survival analyses according to activated cancer-associated fibroblasts in the TCGA dataset (617 cases)

| **Disease-specific survival** | **Univariate[a]** | **Multivariate[b]** | **HR** | **95% CI** | |
|---|---|---|---|---|---|
| aCAFs (absence versus presence) | **<0.001** | **<0.001** | 8.585 | 3.503 | 21.039 |
| Age (≤55 versus >55) | 0.738 | 0.672 | 0.840 | 0.374 | 1.884 |
| Sex (women versus men) | 0.631 | 0.932 | 1.036 | 0.456 | 2.353 |
| T stage (1, 2, 3 versus 4) | **<0.001** | **<0.001** | 7.109 | 3.105 | 16.277 |
| Histological grade (1, 2 versus 3) | **<0.001** | 0.109 | 1.978 | 0.860 | 4.550 |
| LVI (absence versus presence) | **<0.001** | 0.669 | 1.221 | 0.489 | 3.048 |
| PNI (absence versus presence) | 0.086 | 0.852 | 0.920 | 0.384 | 2.202 |
| Tumor necrosis (absence versus presence) | 0.347 | 0.534 | 0.693 | 0.218 | 2.204 |
| **Overall survival** | | | | | |
| aCAFs (absence versus presence) | **<0.001** | **<0.001** | 3.591 | 1.917 | 6.727 |
| Age (≤55 versus >55) | **0.043** | 0.153 | 1.719 | 0.817 | 3.616 |
| Sex (women men) | 0.605 | 0.933 | 1.028 | 0.535 | 1.977 |
| T stage (1, 2, 3 versus 4) | **<0.001** | **<0.001** | 3.835 | 1.864 | 7.890 |
| Histological grade (1, 2 versus 3) | **<0.001** | 0.513 | 1.283 | 0.608 | 2.705 |
| LVI (absence versus presence) | **<0.001** | 0.432 | 1.345 | 0.642 | 2.815 |
| PNI (absence versus presence) | 0.104 | 0.993 | 0.997 | 0.478 | 2.078 |
| Tumor necrosis (absence versus presence) | 0.306 | 0.451 | 0.729 | 0.320 | 1.660 |

T stage by the 8th edition of the American Joint Committee on Cancer.

Histological grade: 1 (well-differentiated), 2 (moderately differentiated), 3 (poorly differentiated) aCAFs: activated cancer-associated fibroblasts, LVI: lymphovascular invasion, PNI: perineural invasion.

[a]Log rank test.

[b]Cox proportional hazard model.
